# Changes in SUMO-modified proteins in Epstein-Barr virus infection identifies reciprocal regulation of TRIM24/28/33 complexes and the lytic switch BZLF1

**DOI:** 10.1371/journal.ppat.1011477

**Published:** 2023-07-06

**Authors:** Carlos F. De La Cruz-Herrera, Michael H. Tatham, Umama Z. Siddiqi, Kathy Shire, Edyta Marcon, Jack F. Greenblatt, Ronald T. Hay, Lori Frappier

**Affiliations:** 1 Department of Molecular Genetics, University of Toronto, Toronto, Canada; 2 Division of Gene Regulation and Expression, School of Life Sciences, University of Dundee, Dundee, United Kingdom; 3 Donnelly Centre, University of Toronto, Toronto, Canada; University of Florida, UNITED STATES

## Abstract

SUMO modifications regulate the function of many proteins and are important in controlling herpesvirus infections. We performed a site-specific proteomic analysis of SUMO1- and SUMO2-modified proteins in Epstein-Barr virus (EBV) latent and lytic infection to identify proteins that change in SUMO modification status in response to EBV reactivation. Major changes were identified in all three components of the TRIM24/TRIM28/TRIM33 complex, with TRIM24 being rapidly degraded and TRIM33 being phosphorylated and SUMOylated in response to EBV lytic infection. Further experiments revealed TRIM24 and TRIM33 repress expression of the EBV BZLF1 lytic switch gene, suppressing EBV reactivation. However, BZLF1 was shown to interact with TRIM24 and TRIM33, resulting in disruption of TRIM24/TRIM28/TRIM33 complexes, degradation of TRIM24 and modification followed by degradation of TRIM33. Therefore, we have identified TRIM24 and TRIM33 as cellular antiviral defence factors against EBV lytic infection and established the mechanism by which BZLF1 disables this defence.

## Author summary

The activity of many proteins is controlled by modification by the small ubiquitin-like modifier (SUMO), and some of these proteins are part of antiviral responses that limit herpesvirus infections, including Epstein-Barr virus (EBV). While often asymptomatic, EBV is clinically relevant as a causative agent in several types of cancer as well as multiple sclerosis. To identify regulators of EBV, we performed a large-scale analysis of SUMO-modified proteins that change in modification or abundance in response to reactivation of EBV from latent to lytic infection. Interesting findings include changes in three related proteins (TRIM24/TRIM28/TRIM33) known to form a complex. Further studies showed that these proteins inhibit EBV lytic infection by suppressing the expression of the first lytic protein, BZLF1. However, BZLF1 was found to interact with and destabilize TRIM24 and TRIM33, disabling their antiviral function. Therefore, we have identified a new cellular defense against EBV and a mechanism by which this virus disables the cellular response.

## Introduction

Epstein-Barr virus (EBV) is a herpesvirus that persistently infects the majority of the world’s population and is the causative agent of infectious mononucleosis. EBV infection is also causatively associated with several types of lymphomas, nasopharyngeal carcinoma and 10% of gastric carcinoma, accounting for ~200,000 new cancer cases and ~140,000 deaths per year [1,2]. In addition, strong evidence has accumulated linking EBV infection to multiple sclerosis [3,4]. Like all herpesviruses, life-long infection occurs due to the ability of EBV to alternate between latent and lytic modes of infection. During latency, EBV gene expression is restricted to a few proteins which modulate the cellular environment to promote cell immortalization and mediate the replication and stable maintenance of EBV episomal genomes without virion production [5,6]. Lytic infection involves the ordered expression of ~70 additional proteins which together manipulate many cellular processes, including innate immune responses, cell cycle progression and DNA damage responses, ultimately leading to production of linear dsDNA genomes and their packaging into infectious virions [5,7].

Posttranslational modification of proteins is a rapid mechanism to modulate the functional diversity of the cellular proteome during normal cell function and in response to stress stimuli such as viral infections. Modification by the Small Ubiquitin-like Modifier (SUMO), referred to as SUMOylation, can affect protein stability, localization and protein-protein or protein-nucleic acid interactions. Similar to ubiquitylation, SUMOylation is catalysed by an E1 activating enzyme, E2 conjugating enzyme and E3 ligases, and can be reversed by SUMO-specific proteases. SUMO conjugation typically occurs on lysines found within a SUMO conjugation consensus motif ψKxD/E where ψ is a large hydrophobic residue [8]. Human cells express three SUMO paralogues, SUMO1, SUMO2 and SUMO3. SUMO2 and SUMO3 are considered functionally equivalent due to 97% identity and are generally referred to as SUMO2/3. Although SUMO1 can form SUMO chains, SUMO2/3 do so more readily due to internal SUMO conjugation sites found within a conjugation consensus motif that is not present in SUMO1 [9]. Proteome-wide studies have shown that SUMO1 and SUMO2/3 modify hundreds of proteins and their substrates overlap significantly [10–18]. These studies have also shown that SUMOylation regulates multiple pathways important for viral infection, antiviral responses and oncogenesis, suggesting that it would be an important determinant in EBV infection and associated diseases. The importance of SUMOylation in regulating herpesvirus infections has been well documented for herpes simplex virus 1 (HSV-1). In particular, promyelocytic leukemia (PML) proteins and PIAS1, PIAS2β and PIAS4 SUMO ligases can associate with and repress HSV-1 genomes dependent on SUMOylation. To counter this, the HSV-1 protein ICP0, which has SUMO-targeted ubiquitin-ligase activity, can catalyse their ubiquitin-mediated degradation [19–22].

The interplay between EBV infection and SUMOylation is less well defined, but several observations suggest that modulation of SUMOylation is important for regulating EBV infections. For example, latent membrane protein 1 (LMP1) induces SUMOylation in latent infection through a variety of mechanisms including increasing SUMO expression, stimulating the activity of the Ubc9 SUMO E2 enzyme, and inhibiting the SUMO protease activity of SENP2 [23–25]. This increases net SUMOylation and appears to promote latency, at least in part through SUMOylation of TRIM28 (also called TIF1β or KAP1), which increases its repression of the lytic immediate early promoters and lytic replication origin [26]. Progression through the lytic cycle also appears to involve modulation of SUMOylation, as several EBV lytic proteins have been found to affect SUMOylation [27]. Immediate early transcriptional activator RTA (also called BRLF1) induces proteasomal degradation of SUMOylated proteins [27]. In contrast, the early lytic protein SM (also called EB2) and the late lytic protein BGLF2 both induce SUMOylation [27]. SM has properties of a SUMO ligase and preferentially induces SUMO1 modification of cellular proteins [27]. BGLF2 upregulates the level of free SUMO by interfering with the function of let-7 miRNA in silencing SUMO transcripts [28]. These findings suggest that net down-regulation of SUMOylation is important to initiate lytic infection but that SUMOylation of specific targets might be important for progression through the lytic cycle.

Insights into cellular proteins that restrict viral infections and are modulated by viral proteins have come from profiling changes in SUMO-modified proteins during HSV1 and influenza virus infection [11,12,18]. To gain a more comprehensive understanding of changes in SUMOylation that promote and regulate EBV infection, we used a SUMO proteomics approach to identify proteins that change in SUMO1 and SUMO2 modifications in response to EBV reactivation from latent to lytic infection. This identified changes in all members of the TRIM24/TRIM28/TRIM33 complex, with increased SUMOylation of TRIM33 and degradation of TRIM24 being among the most notable responses to EBV reactivation. We showed that both TRIM24 and TRIM33 repress expression of the EBV BZLF1 lytic switch gene and that BZLF1 binds TRIM24 and TRIM33 to disrupt TRIM24/TRIM28/TRIM33 complexes. Therefore, we identified TRIM24/TRIM28/TRIM33 complexes as restriction factors for EBV lytic infection and a mechanism by which BZLF1 disables these complexes.

## Results

### Inhibition of SUMO conjugation promotes EBV lytic infection

**Fig 1:**
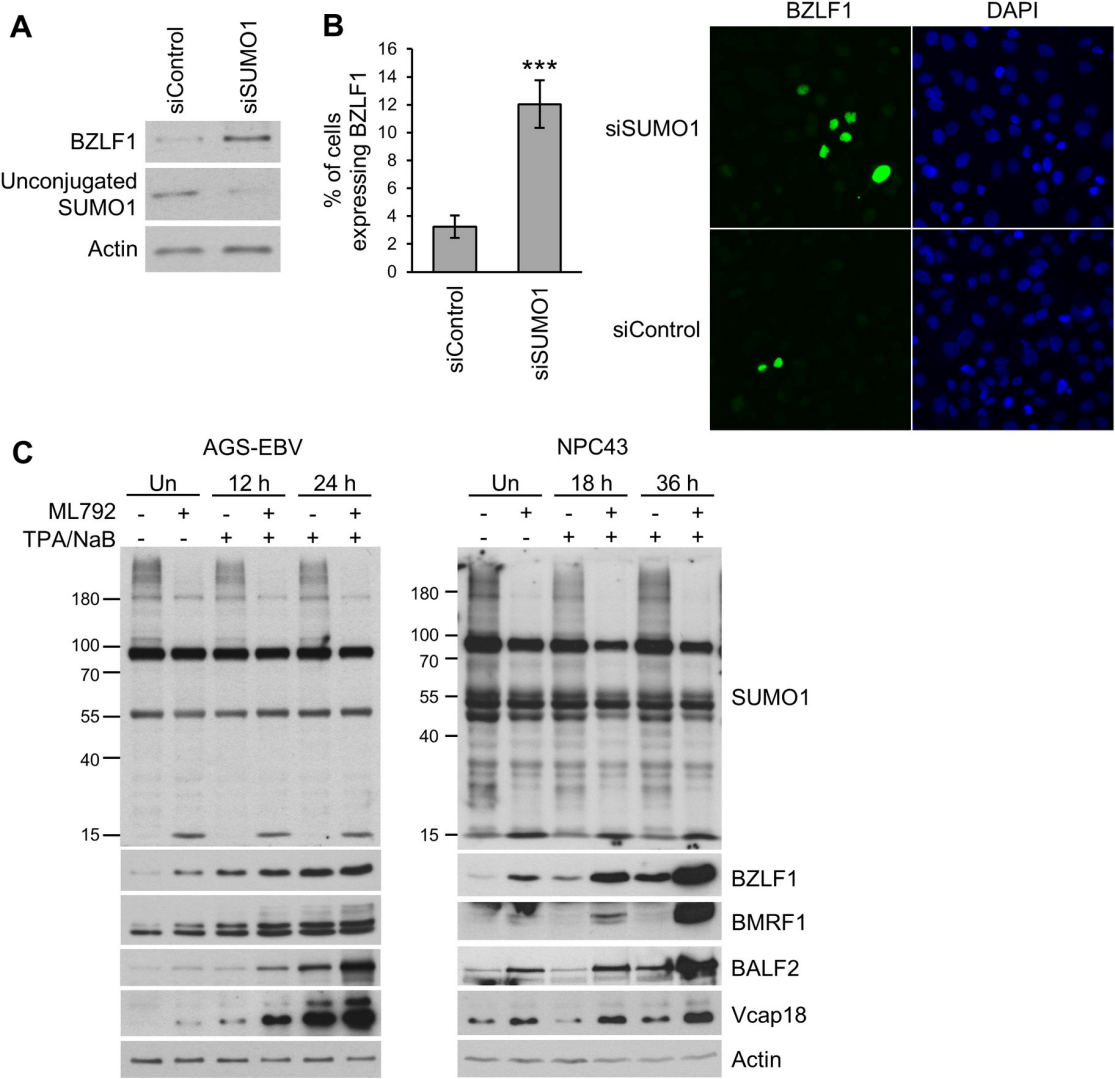
Inhibition of SUMOylation promotes EBV lytic infection. A and B. AGS-EBV cells were treated with siRNA against SUMO1 or negative control siRNA, then analyzed by Western blot with antibodies against BZLF1, SUMO1 and actin (A) or fixed and stained with BZLF1 antibody for IF (B). The percentage of BZLF1 expressing cells was determined from 300 cells in three independent experiments and average values are shown on the graph. C. Western blot analysis of AGS-EBV and NPC43 cells treated with the SUMO E1 conjugating enzyme inhibitor ML792 or DMSO (-) before (Un) or the indicated hours after induction of EBV lytic reactivation with TPA/NaB. Total protein extracts were probed with antibodies against SUMO1, BZLF1, BMRF1, BALF2, Vcap18 and actin.

Modulation of SUMOylation is central to herpesviruses lytic infections, as these viruses encode multiple proteins that either induce SUMOylation or induce the degradation of SUMO-modified host proteins [27,29,30]. To determine how downregulation of SUMO affects EBV reactivation and lytic infection, SUMO1 was silenced in AGS gastric carcinoma cells latently infected with EBV (AGS-EBV), using SUMO1-targeted short interfering RNA (siRNA). Expression of the EBV lytic switch protein, BZLF1, was assessed as a measure of EBV reactivation. Western blot analysis indicated that siRNA-mediated depletion of SUMO1 resulted in higher levels of BZLF1 expression (Fig 1A). Immunofluorescence microscopy revealed that this was largely a consequence of SUMO1 silencing leading to a 4-fold increase in the number of cells expressing BZLF1 (Fig 1B).

We further investigated the importance of SUMOylation in EBV lytic infection by inhibiting the SUMO activating enzyme (SAE) with the specific inhibitor ML792 [31]. AGS-EBV cells and NPC43 EBV-positive nasopharyngeal carcinoma cells were treated with ML792 (or mock treated), then cell lysates were analysed by Western blotting before or at two time points after reactivation of the lytic cycle with 12-O-tetradecanoylphorbol-13-acetate (TPA) and sodium butyrate (NaB). ML792 treatment resulted in higher levels of immediate early (BZLF1), early (BMRF1, BALF2), and late (Vcap18) EBV proteins at all time points in both AGS-EBV and NPC43 cells (Fig 1C), indicating that an active SUMO conjugation system is generally inhibitory to EBV reactivation and lytic infection. These results suggest that SUMOylation of cellular or viral proteins restricts EBV reactivation.

### Generation of AGS-EBV-SUMO cells for SUMO proteomics analysis

**Fig 2:**
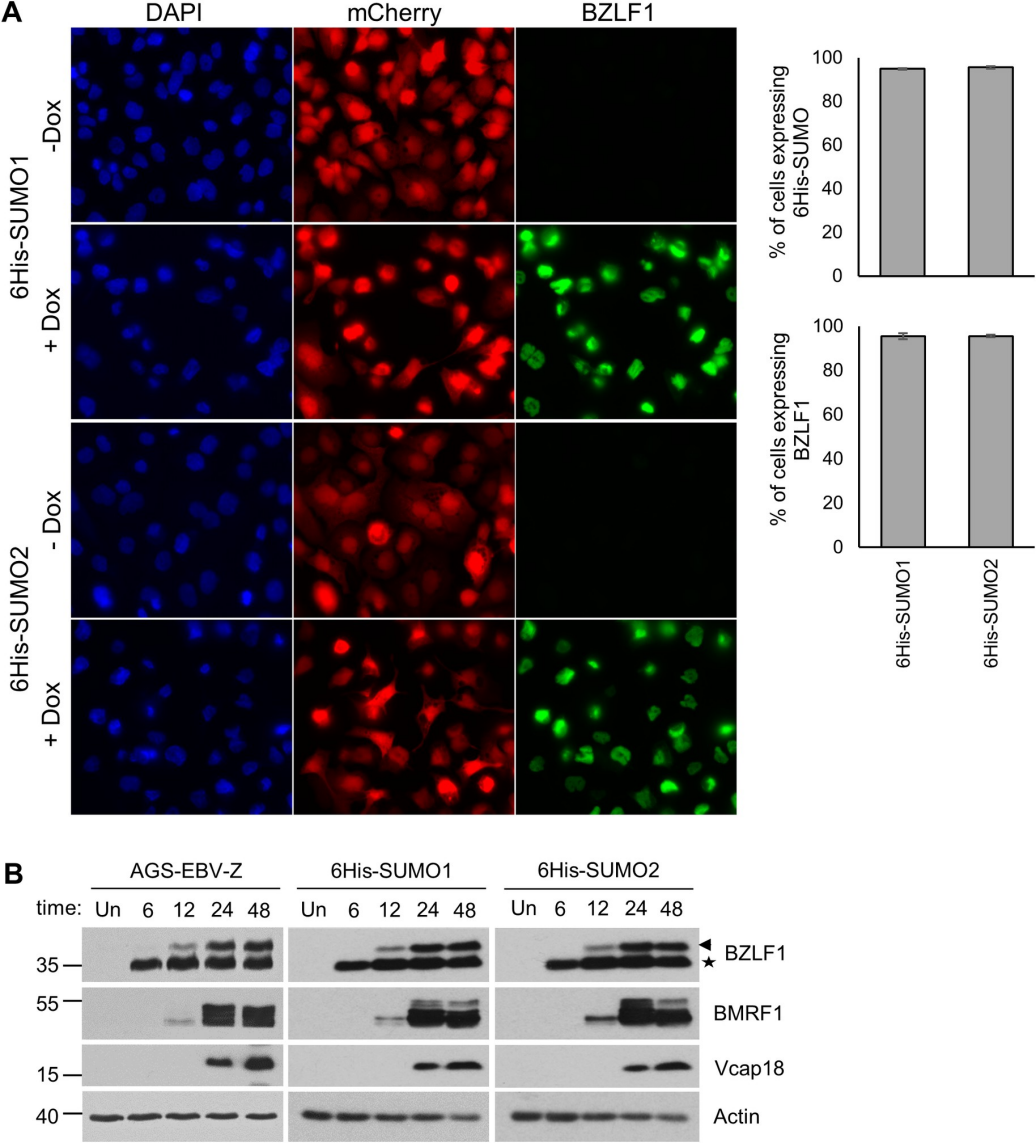
Characterization of cell lines used for the proteomic experiments. A. IF of the 6His-SUMO1 or 6His-SUMO2 cells showing most express both the mCherry marker linked to SUMO and the lytic switch protein BZLF1 (detected with anti-BZLF1 antibody) after dox induction. B. Comparison of expression of EBV lytic proteins before (Un) or the indicated hours after dox-induction of the lytic cycle in AGS-EBV-Z cells with and without integrated 6His-SUMO1 or 2. Arrow and star indicate endogenous and integrated BZLF1, respectively.

To gain insight into the SUMOylated proteins that might regulate EBV reactivation and lytic infection, we set out to profile changes in SUMO modifications of cellular proteins that occur in response to EBV reactivation. Since reactivation of latently infected EBV cells is inefficient using standard methods (including TPA/NaB), we used AGS-EBV cells with doxycycline (dox)-inducible BZLF1 (AGS-EBV-Z) that we previously generated and showed to efficiently reactivate after dox addition [32]. AGS-EBV-Z cells were engineered to stably express 6His-SUMO-mCherry proteins with C-terminal TGG to KGG mutations (SUMO1^T95K^ and SUMO2^T90K^), which facilitate mapping by mass spectrometry (MS) of SUMO modifications, due to a diagnostic diGly (GG) motif that remains attached to target proteins after Lys-C digestion [14,15]. The C-terminally fused mCherry is cleaved by endogenous SUMO proteases to expose the C-terminal GG motif for conjugation and serves as a marker for cells expressing the exogenous SUMO. AGS-EBV-Z-6His-SUMO cells were first analyzed to ensure that expression of dox-induced BZLF1 and progression of the EBV lytic cycle was unaffected by the integration of the 6His-SUMOs. As shown in Fig 2A, the cells consistently expressed 6His-SUMO1-mCherry or 6His-SUMO2-mCherry proteins and, after dox treatment, most (~95%) also expressed BZLF1. In addition, Western blotting showed that expression of BZLF1 (dox-induced and endogenous), BMRF1 (early) and Vcap18 (late) viral proteins in the 6His-SUMO cells mirrored that of the parental AGS-EBV-Z cells (Fig 2B). Together, these results indicate that expression of 6His-SUMO1^T95K^ or 6His-SUMO2^T90K^ did not affect the reactivation and progression of the EBV lytic cycle in AGS-EBV-Z cells.

### Changes in SUMO1- and SUMO2-modified proteins upon EBV lytic infection

**Fig 3:**
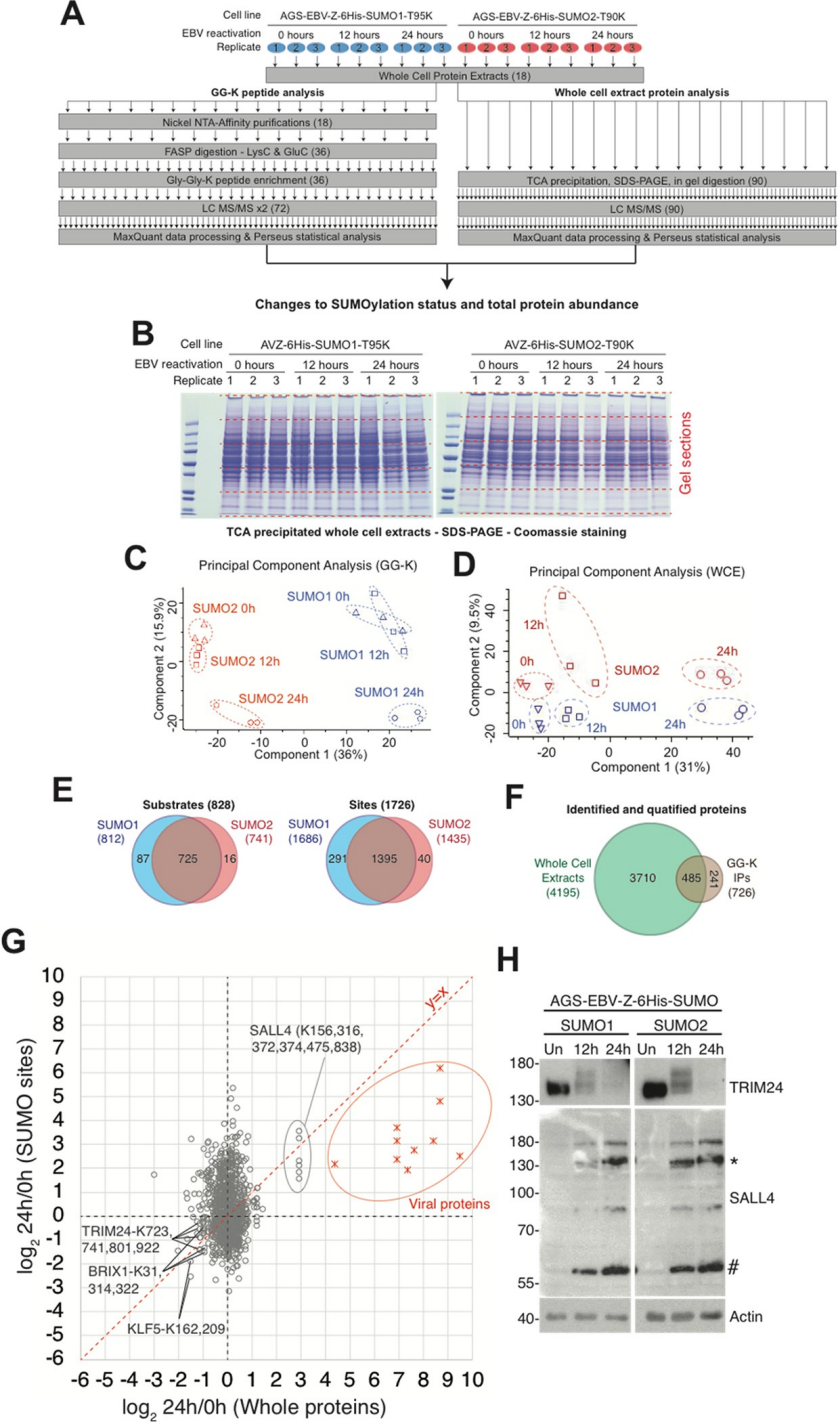
Overview of the proteomics experiments. A. The label-free proteomics experimental design to monitor changes to cellular protein abundance and the SUMOylation landscape during EBV reactivation. B. Coomassie-stained gel of the whole cell extract triplicate data for total proteome analysis. C + D Principal component analysis of proteomic data for both GG-K peptide intensity and whole protein intensity for the SUMOylation analysis (C) and the total proteome analysis (D). E. Summary of substrates and sites identified based on having at least one intensity reported in at least one MS run associated with SUMO1 or SUMO2 derived samples. F. Numbers of proteins identified and quantified from the whole cell lysate analysis, and the degree of overlap with the SUMO substrates identified. G. Relationship between total protein abundance change (x-axis) and GG-K peptide abundance change (y-axis) during 24h EBV reactivation. x- and y-axes show average of SUMO1 and SUMO2 ratios. y = x line is shown in red. Sites in viral proteins have red markers, cellular proteins in grey. Selected sites are indicated. H. Examples of two proteins (SALL4 and TRIM24) found to be altered in levels upon EBV reactivation. Western blots were performed on the cell lysates used for the mass spectrometry analyses using antibodies against TRIM24 and SALL4. Positions of SALL4A (*) and SALL4B (#) are indicated.

The AGS-EBV-Z-6His-SUMO1 and AGS-EBV-Z-6His-SUMO2 cells were used to monitor changes in SUMO-modified proteins that occur when EBV reactivates from the latent to the lytic cycle. The cells were either left untreated (latent infection) or harvested 12 or 24 hours after dox treatment to induce the lytic cycle. 6His-SUMO conjugated proteins were isolated from cell lysates under denaturing conditions on metal chelating resin followed by enzymatic digestion with LysC and GluC, GG-K peptide immunoprecipitation, and then analysed by mass spectrometry (Fig 3A). In parallel, protein level changes were also monitored by MS analysis of peptides generated from in gel tryptic digestion of protein extracts from whole cell lysates (Fig 3A and 3B). Three independent experiments were performed per condition. Principal component analysis of MS protein intensity data showed that replicates of the same timepoint and the same cell line clustered together for both the GG-K peptides and the whole proteins (Fig 3C and 3D). From GG-K peptides, 1726 SUMO1 or SUMO2 sites were identified from 828 proteins (Fig 3E), of which 1424 sites in 726 proteins were identified in all three replicates of at least one experimental condition and were carried forward for statistical analysis (see S1 Table for details). Consistent with previous large-scale SUMO proteomics studies, many proteins were found to have multiple modification sites. Particularly highly modified proteins include ZNF106 with 18 sites, RANBP2 with 17 sites, NPM1, RSL1D1 and SAFB2 with 12 sites, KRT8, NOLC1, NOP58, TRIM28, and ZNF451 with 11 sites, AHNAK, BRD7, DKC1, RSF1 and WIZ with 9 sites, and BCLAF1, MBD1 and MGA with 8 sites (S1 Table). Importantly, 485 of the SUMO substrates were also quantified in crude protein extracts (Fig 3F), which allowed comparisons between protein level and SUMOylation site level changes. There was poor correlation between the average of the SUMO1 and SUMO2 site-level data after 24h EBV reactivation, and the corresponding protein-level data from crude extracts (Fig 3G), confirming that most changes in GG-K peptide abundance could not be explained by altered total protein abundance. However there were exceptions, including a group of viral proteins found to be SUMO modified (discussed below) and the cellular zinc-finger transcription factor SALL4 (containing six sites) which showed an ~8-fold increase in abundance upon EBV reactivation for 24 hours (Figs 3G and S1A). Conversely, the ribosome biogenesis regulator BRIX1 (3 sites), the transcription factor KLF5 (2 sites) and the ubiquitin E3 ligase TRIM24 (4 sites) all roughly halved in protein abundance over the same period, likely explaining their apparent reduction in SUMOylation (Figs 3G and S1B). These results were confirmed by Western blotting for SALL4 and TRIM24 (Fig 3H). The levels of several other SUMO-modified proteins were confirmed to remain unchanged (S2A Fig). We also verified that the decrease in TRIM24 and increase in SALL4 after EBV reactivation was not a direct effect of the dox inducible system, as these proteins were unaffected when dox was added to AGS cells containing the empty TRIPZ construct (S2B Fig) or TRIPZ with a dox-inducible GFP (AGS-GFP; S2C Fig).

**Fig 4:**
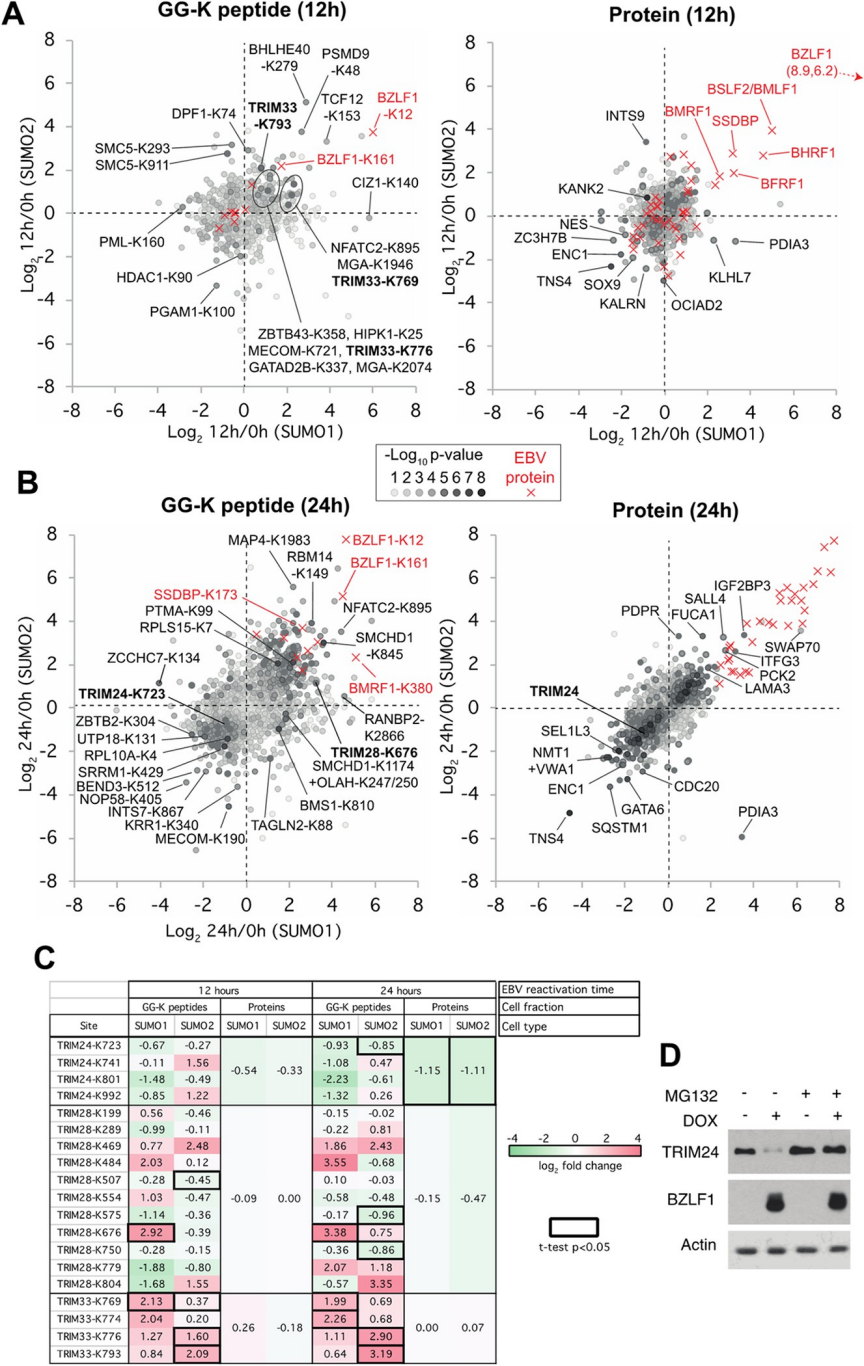
Proteomics data summary. A + B. Charts indicating changes in abundance to SUMO1 (x-axis) and SUMO-2 (y-axis) GG-K peptides (left) and cellular proteins (right) derived from whole cell extracts from the 6His-SUMO1 cells (x-axis) and 6His-SUMO2 cells (y-axis) in response to 12 hours (A) or 24 hours (B) EBV reactivation. Data derived from viral proteins are indicated by red markers and cellular proteins are grey, with the shade indicating the statistical significance of the change (see key) derived from the sum of the two -log_10_ p-values for the x and y-axis data. Selected sites and proteins with large deviation from 0 combined with high significance are indicated. C. Tabular summary of the SUMO1 and SUMO2 site-level data and total protein level data for TRIM24, TRIM28 and TRIM33. Cells are coloured by fold change with a thick border indicating statistical significance (p<0.05). D. AGS-EBV-Z cells were treated with dox (or left untreated) with and without MG132 for 12 hours as indicated. Western blots were then performed using antibodies against TRIM24, BZLF1 and actin.

An overview of the GG-K peptide data at 12 hours after EBV reactivation (Fig 4A left) shows mostly modest ratios, with very few SUMO sites showing statistically significant changes. Only 57 SUMO1 sites and 107 SUMO2 sites significantly changed (p<0.05) when comparing latent infection (0 hours) with 12 hours post-reactivation (S1 Table). This increased to 278 SUMO1 and 321 SUMO2 sites with significant changes 24 hours post-reactivation (S1 Table and Fig 4B, left). Similarly, the whole cell extract analysis showed that changes in the total proteome were greater 24 hours after EBV reactivation than at 12 hours (Fig 4A and 4B, right), although at both times the changes to the SUMOylome were generally greater than total protein changes (compare left with right charts in Fig 4A and 4B).

SUMO modifications at most sites did not change significantly in response to lytic infection. For example, some highly SUMO-modified proteins, including BCLAF1, TCOF1, SAFB and NOLC1, showed changes to only a small proportion of their total number of SUMO sites (S1C Fig). However, there were also groups of proteins that consistently became SUMOylated or deSUMOylated during EBV reactivation. For example, NOP58, BRD7, BRD8, PML, RBM25, BEND3, RSL1D1, TOPORS, ZBTB2 and ZBTB25 all showed decreased SUMO1 and SUMO2 conjugation (S1D Fig). In contrast, RBBP4, NSMCE2, NSMCE4A, RBM14, NFATC2, CHD4, GATAD2A, GATAD2B, SERBP1, PARP1, SAMHD1 and SP100 had increased SUMOylation (S1E Fig). Strikingly, 18 sites of SUMOylation were detected from SUMO1, SUMO2 and SUMO3 themselves, all of which increased upon EBV reactivation (S1E Fig), suggesting increased SUMO polymerisation is a consequence of lytic EBV infection. We also identified some proteins with changes specific to SUMO1 or SUMO2. For example, a group of mainly nuclear pore proteins, including RANGAP1, RANBP2, RGPD1 and RGPD4, responded to lytic infection by gain of SUMO1 but not SUMO2 modification (S1F Fig). In contrast, ZNF451 and SMC5, which was recently shown to restrict lytic infection by EBV [33] and other herpesviruses [34,35], showed increased SUMO2-specific modification (S1G Fig). Finally, there was a small group of proteins, including MGA, TRIM28 and TRIM33, that displayed an unusually early response to EBV reactivation, as well as having SUMO paralogue-specific gains and losses at different sites (Figs 4A and S1H). TRIM28 and TRIM33 are known to form a trimeric complex with TRIM24, which itself was found (above) to reduce in abundance in lytic infection (Fig 3G and 3H). All three of these TRIM proteins experienced some form of alteration in response to EBV reactivation, with TRIM28 and TRIM33 exhibiting increased SUMO modifications as early as 12 hours post-reactivation and TRIM24 levels significantly decreasing by 24 hours (Fig 4C).

An overall view of EBV-affected cellular SUMOylation was generated by aggregating all SUMOylation changes for all sites into a single metric of Log_2_ fold SUMO change per hour and overlaying this onto a STRING network of all identified SUMO substrates (S3A Fig). Generally, there is a net loss of SUMO from zinc-finger proteins and those involved in mRNA splicing and translation functions, while many proteins involved in chromatin structure/transcriptional regulation, and protein post-translational modification showed increased SUMOylation. Most strikingly, members of the NuRD complex, nuclear pore complex and actin-binding proteins displayed net SUMOylation increases (S3B to S3D Fig).

**Fig 5:**
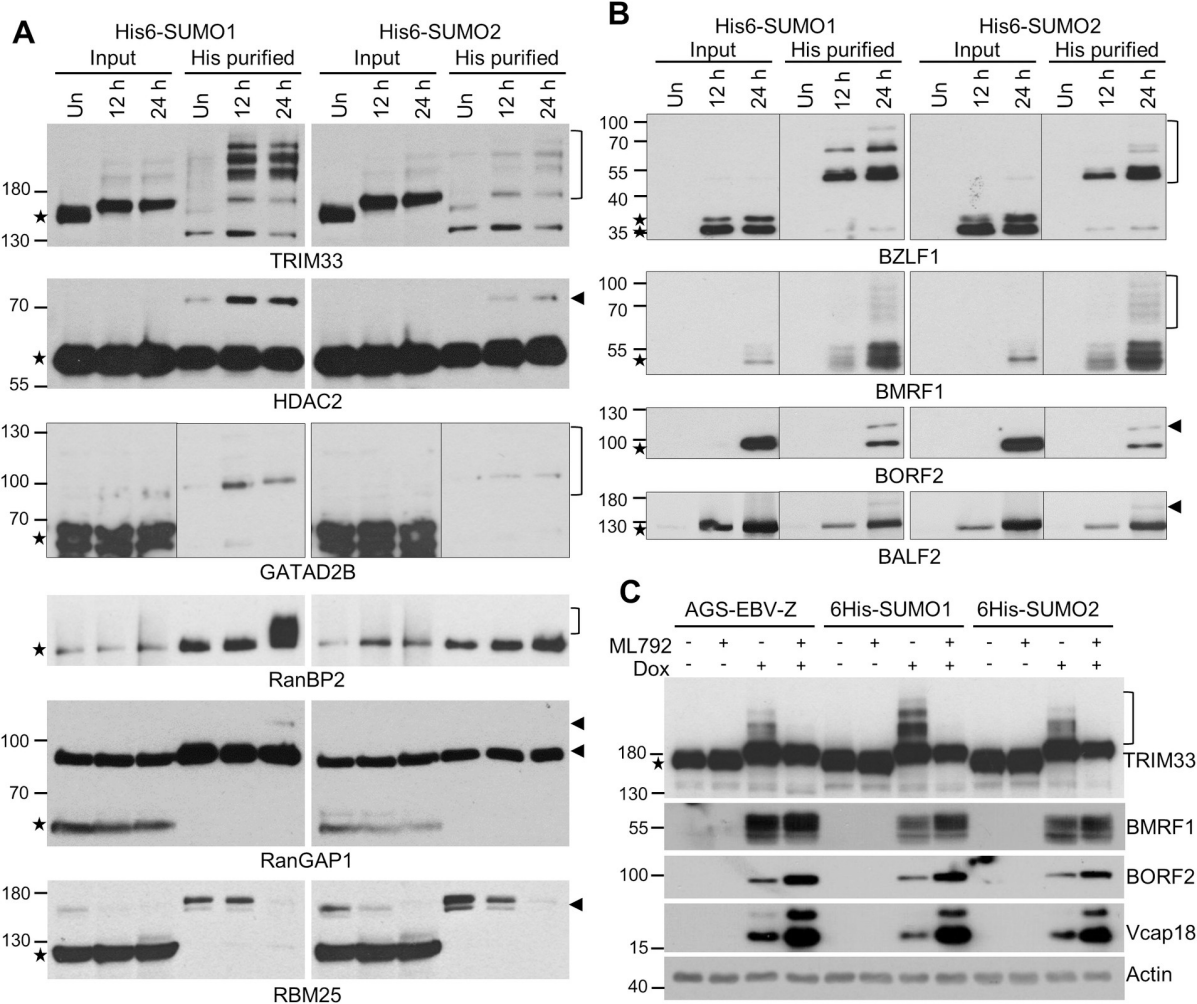
Verification of changes in SUMOylation in selected cellular and viral proteins. A and B. Whole cell lysates from AGS-EBV-Z-6His-SUMO1 and AGS-EBV-Z-6His-SUMO2 cells before (Un) or 12 or 24 hours after dox induction were analysed by Western blotting using the indicated antibodies. Examples of cellular proteins with altered SUMOylation after EBV reactivation (A) and EBV proteins with SUMO modifications (B) are shown. Stars indicate nonmodified proteins. Arrowheads and brackets indicate SUMO conjugated proteins. C. The indicated cell lines were treated with ML792 or left untreated then reactivated with dox for 24 hours or left untreated. Whole cell lysates were then analysed by Western blotting and probed for EBV lytic proteins (BORF2, BMRF1, Vcap18) and TRIM33.

To confirm the changes in SUMOylation detected by mass spectroscopy, selected proteins were examined by Western blotting both in whole cell lysates from 6His-SUMO cells and after recovery of 6His-SUMO conjugated proteins on nickel resin (Fig 5A). After 6His-purification, all these proteins showed changes in SUMO1 and/or SUMO2 modifications consistent with the mass spectrometry results.

### Identification of EBV proteins that are modified by SUMO

In addition to cellular proteins that are SUMOylated, our proteomic analysis identified seven viral proteins that were SUMO-modified in EBV lytic infection. These are transcriptional activators BZLF1 and BRLF1, DNA polymerase processivity factor BMRF1, mRNA export protein SM, DNA binding protein BALF2, ribonucleotide-reductase large subunit BORF2, and viral replication regulator LF2. Of these, BZLF1, BRLF1, BMRF1, SM and LF2 have been previously reported to be SUMO-modified [27,36–39]. A summary of EBV proteins that are modified by SUMO and the sites modified by SUMOylation and phosphorylation are shown in S2 Table. SUMOylation of selected EBV proteins (BZLF1, BMRF1, BORF2, BALF2) was verified by nickel purification of 6His-SUMO conjugated proteins from AGS-EBV-Z-6His-SUMO1 and 6His-SUMO2 cells reactivated to lytic infection followed by Western blotting with specific antibodies (Fig 5B).

### Alterations of TRIM24 and TRIM33 in response to EBV reactivation

Our proteomics experiments identified significant changes to the overall protein levels of TRIM24 (TIF1α) and the SUMOylation status of TRIM28 (TIF1β or KAP1) and TRIM33 (TIF1γ), which are known to interact with each other and have roles in transcriptional repression [40,41]. TRIM28 showed an unusually diverse, site-specific range in SUMOylation responses during EBV lytic infection, with increased SUMO1 modification at Lys676 and reduced SUMO2 modification at lysines 575 and 750. The reduction in TRIM24 protein levels followed a mobility shift, suggesting its modification (Fig 3H). TRIM24 is known to be a labile protein regulated by ubiquitylation and proteasomal degradation [40,42], prompting us to ask if the loss of TRIM24 in EBV lytic infection is due to proteasomal degradation. Consistent with this hypothesis, loss of TRIM24 in reactivated AGS-EBV-Z cells was prevented when the cells were treated with the MG132 proteasomal inhibitor (Fig 4D).

EBV lytic infection triggered a large increase in TRIM33 SUMO modifications, to the point that shifted bands that may correspond to SUMOylated proteins became detectable in whole cell lysates without enrichment of SUMOylated proteins (Figs 5A and S2A). We investigated which of these bands were due to SUMOylation, by comparing AGS-EBV-Z-6His-SUMO1 and AGS-EBV-Z-6His-SUMO2 cell lysates before and after reactivation in the presence and absence of the ML792 SUMOylation inhibitor (Fig 5C). After reactivation, the predominant TRIM33 band appeared to undergo a small shift upwards in the gel that was unaffected by ML792, suggesting it was not SUMO-dependent. However, the additional slower migrating bands triggered by reactivation were abrogated by ML792 treatment, indicating that they are SUMOylated products. The same results were seen in the 6His-SUMO expressing cells and parental AGS-EBV-Z cells lacking 6His-SUMO, confirming that TRIM33 SUMOylation is not caused by the overexpression of 6His-SUMOs (Fig 5C). We also verified that the observed modified forms of TRIM33 were not a result of the dox-inducible system, as shifted forms of TRIM33 were not detected when dox was added to AGS cells containing empty TRIPZ or dox-inducible GFP (S2B and S2C Fig, respectively).

**Fig 6:**
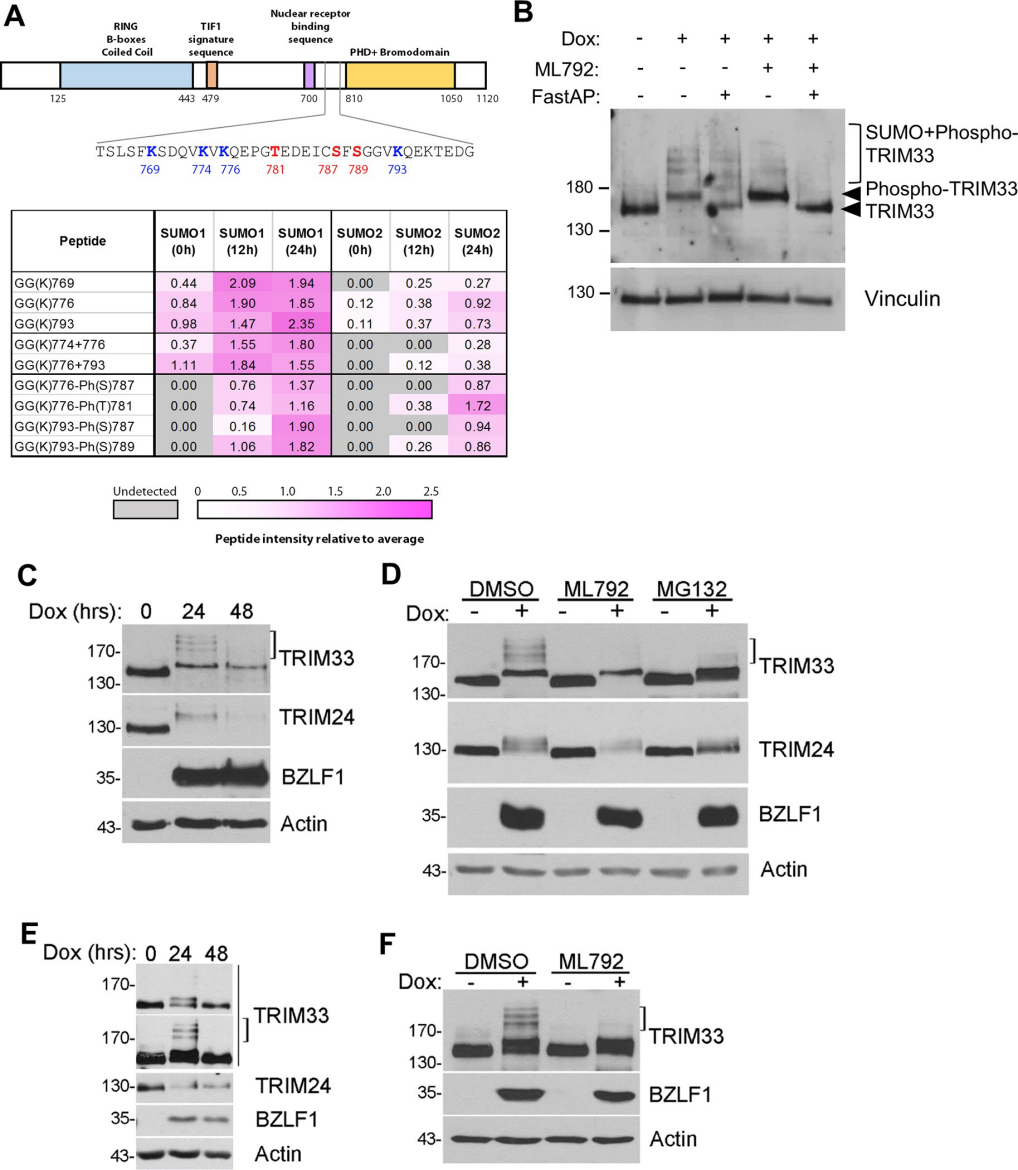
SUMOylation and phosphorylation of TRIM33 and verification of TRIM24 loss in NPC43 and Akata cell infections. A. Upper panel: Schematic of TRIM33 showing domain structure and sites of SUMOylation and phosphorylation identified in the proteomics experiment. Lower Panel: Summary of MS peptide intensity data for the indicated TRIM33 modifications. Mono-SUMOylated peptides, di-SUMOylated peptides, and peptides co-modified by SUMO and phospho moieties are grouped. Intensity in each sample relative to the average intensity across all samples is indicated and coloured according to the scale shown below (values represent the average of three replicates per condition). Peptides not detected in a particular sample are shown in grey. Cell line and duration of EBV reactivation are shown in the headers. B. AGS-EBV-Z cells were treated with dox (or left untreated) with and without ML792 for 12 hours as indicated. Whole cell lysates were incubated with or without FastAP phosphatase, followed by Western blotting with TRIM33 and vinculin antibodies. C. NPC43-Z cells were treated with dox for 0, 24 or 48 hours, then whole cell lysates were analysed by Western blotting using TRIM33, TRIM24, BZLF1 and actin antibodies. The bracket marks the position of bands corresponding to SUMO-modified TRIM33. D. NPC43-Z cells were treated with dox for 12 hours (or left untreated) in the presence of DMSO (negative control), ML792 or MG132 as indicated, and the lysates were anlaysed as in C. E. Akata-Z cells were treated with dox for 0, 24 or 48 hours, then whole cell lysates were analysed by Western blotting using TRIM33, TRIM24, BZLF1 and actin antibodies. Two exposures are shown for TRIM33, with the phospho shift evident in the lighter (top) exposure and the SUMO-modified forms seen in darker exposure and marked by the bracket. F. Akata-Z cells were treated with dox for 24 hours (or left untreated) in the presence of DMSO (negative control) or ML792, and the lysates were anlaysed as in E.

More detailed examination of the mass spectrometry data showed that all TRIM33 SUMO sites map to the same region, between amino acids 769 and 793 (Fig 6A, upper panel). Notably, many of these were identified by peptides carrying two SUMO moieties simultaneously or SUMO modified peptides co-modified with phosphorylation. Both findings are relatively rare. Two reasons make the detection of doubly-SUMOylated peptides unusual in this experiment. Firstly, low occupancy of lysine SUMOylation reduces the likelihood of finding two adjacent lysines simultaneously modified by SUMO. Secondly, endogenous SUMOs can compete with the exogenous T->K SUMO mutants for attachment to lysines, and will ‘dilute’ the GlyGly-K signal monitored in this study. For these reasons it is reasonable to assume peptides carrying more than one GlyGly-K adduct are indicative of regions of proteins with relatively high levels of SUMOylation. Notably, less than 2% of all SUMO modified peptides detected in this study carried more than one GlyGly-K. Only 9% of all GlyGly-K-containing peptides were found to be co-modified by phosphorylation, which is an indication of extensive proximal phosphorylation. In total, 90 proteins were detected with SUMO/phospho co-modified peptides, including TRIM24, TRIM33 and TRIM28 as well as the viral proteins BZLF1 and BMRF1 (S1 and S2 Tables). TRIM33 phosphorylations were detected on T781, S787 and S789, and these SUMO/phospho peptides were only detected in the reactivated EBV samples and not in latent infection (Fig 6A, lower panel). Therefore, TRIM33 is unusual in that it is heavily modified by both SUMO and phosphorylation in response to EBV reactivation. To confirm this, the migration of TRIM33 was examined before and after reactivation of AGS-EBV-Z cells (with dox) in the presence or absence of the SUMOylation inhibitor ML792, and samples of the lysates were treated with FastAP phosphatase (Fig 6B). Western blots showed that the shifted form of TRIM33 that was not due to SUMOylation migrated as unmodified TRIM33 after phosphatase treatment, confirming that this band is due to phosphorylation of TRIM33.

Next we wanted to determine if the changes we observed in TRIM24 and TRIM33 upon EBV reactivation were conserved in other EBV-positive cells. To this end, we integrated a dox-inducible BZLF1 cassette into NPC43 nasopharyngeal carcinoma cells [43] (NPC43-Z) and Akata B cells (Akata-Z), both of which naturally contain EBV. Upon reactivation of NPC43-Z cells with dox, both TRIM33 and TRIM24 exhibited shifted forms, consistent with those seen in AGS-EBV-Z cells, and TRIM24 (and to a lesser degree TRIM33) decreased in abundance (Fig 6C). The decrease in protein levels was inhibited by MG132, consistent with proteasomal degradation (Fig 6D). Treatment with ML792 verified that the higher molecular weight forms of TRIM33 were SUMO-modified but the lowest shifted form was not, consistent with the efficient phosphorylation of TRIM33 as we observed after reactivation in AGS-EBV-Z cells. Similarly, reactivation of Akata-Z cells resulted in loss of TRIM24 and multiple shifted forms of TRIM33 (Fig 6E; TRIM33 phosphoshift seen in the light exposure and SUMO shifts in the dark exposure), with the higher forms being inhibited by ML792 (Fig 6F). Effects of MG132 could not be assessed due to toxicity to Akata cells. Therefore, the induction of TRIM33 phosphorylation and SUMOylation and degradation of TRIM24 occurs in response to EBV reactivation in multiple cell backgrounds.

### Identification of TRIM24 and TRIM33 as restriction factors for EBV lytic infection

**Fig 7:**
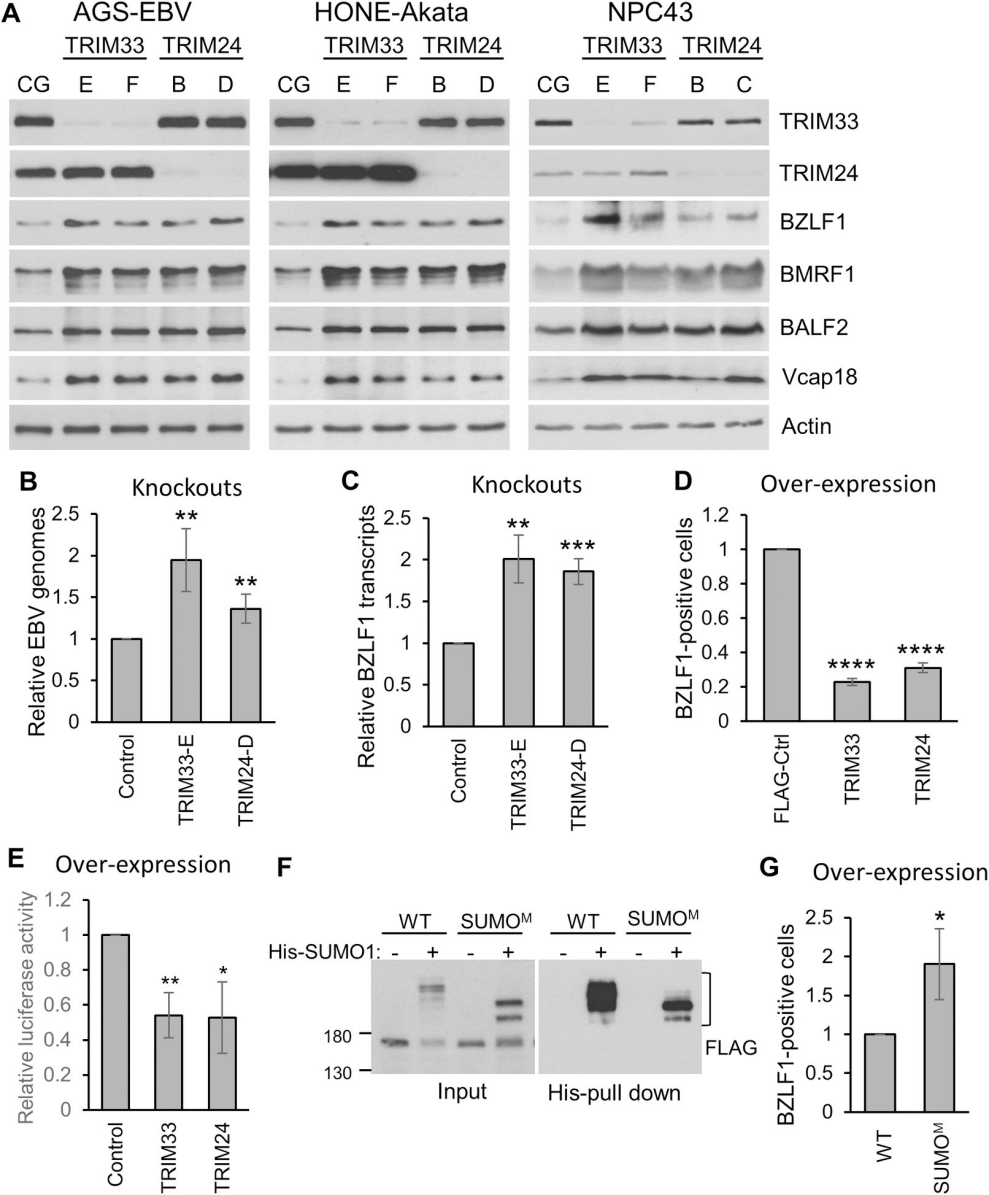
TRIM33 and TRIM24 suppress EBV reactivation. A. TRIM33 or TRIM24 was knocked out using CRISPR-Cas9 in pools of AGS-EBV, HONE-Akata and NPC43 cells with two different guide RNAs (E and F for TRIM33; B and C or D for TRIM24) and compared to a negative control guide RNA (CG) targeting the Adeno-Associated Virus Integration Site 1 (AAVS1). Western blots were performed on whole cell lysates with antibodies against TRIM33, TRIM24, four EBV proteins (BZLF1, BMRF1, BALF2, Vcap18) and actin. B and C. Total DNA and RNA were extracted from AGS-EBV cells in A and EBV genome amplification (B) and BZLF1 transcripts (C) were quantified by qPCR. D. AGS-EBV cells were transfected with plasmids expressing a negative control FLAG-tagged protein (DDX24) or FLAG-tagged TRIM33 or TRIM24, then stained for BZLF1 and FLAG and imaged by IF. For each of the transfected plasmids, the percentage of FLAG-positive cells expressing BZLF1 was determined in 100 cells in three independent experiments. E. AGS cells were co-transfected with pZp-luc (firefly luciferase) and pRL-promotorless (renilla) reporter plasmids along with FLAG-TRIM33 or FLAG-TRIM24 expression plasmid or empty plasmid control and harvested 24 hours post-transfection. Firefly and renilla luciferase levels were quantified and luciferase values were normalized to renilla for three independent experiments. F. WT and SUMO mutant (SUMO^m^) TRIM33 were expressed in 293T cells with or without 6His-SUMO1 and 10% of cell lysates were analysed as input samples. SUMO-modified proteins were isolated from the remaining samples on metal chelating resin (His pull down). Samples were analysed by Western blotting using anti-FLAG antibody. G. The same experiments as in D except that WT TRIM33 was compared to the TRIM33 SUMO^m^. All graphs show average values with standard deviation where * = 0.01<P≤0.05, ** = 0.001< P ≤ 0.01, *** = 0.0001<P≤0.001.

The alteration in abundance or SUMOylation status of TRIM24, TRIM28 and TRIM33 in response to EBV reactivation suggests that these proteins might play a role in restricting EBV lytic infection. While TRIM28 has been previously shown to repress BZLF1 expression [26,44], potential roles of TRIM24 and TRIM33 in restricting EBV infection have not been investigated. We knocked out TRIM24 or TRIM33 in AGS-EBV cells using lentivirus-delivered CRISPR-Cas9 with two different guide RNAs targeting each gene and compared effects on EBV reactivation to a lentivirus-delivered negative control guide RNA. The expression of all EBV lytic proteins examined (including BZLF1) was increased by both of the TRIM24- and TRIM33-targeted guides relative to the control guide (Fig 7A). These knockouts were then made in the EBV-positive HONE1-Akata cell line, as well as in NPC43 nasopharyngeal carcinoma cells that naturally contain EBV [43]. In both cases, knockouts of TRIM24 or TRIM33 resulted in increased expression of EBV lytic proteins including BZLF1 (Fig 7A), suggesting that these proteins repress BZLF1 expression, which would then affect expression of downstream EBV genes.

If TRIM24 and TRIM33 repress BZLF1 expression, we would expect to see a link between their cellular levels and the amplification of the EBV genomes that occurs in EBV lytic infection after early gene expression. To this end, we quantified EBV genomes by qPCR in AGS-EBV cells, comparing cells with the TRIM24 or TRIM33 knockout to the cells with the negative control guide. Knockout of either TRIM24 or TRIM33 significantly increased EBV genome amplification, with TRIM33 having the larger effect, likely because TRIM24 levels would already be low at this point in infection (Fig 7B). We also asked whether TRIM24 or TRIM33 knockout affected the level of BZLF1 transcripts, as expected if these proteins regulated the BZLF1 promoter. Both knockouts increased the level of BZLF1 transcripts, consistent with roles of these protein in repressing BZLF1 expression (Fig 7C).

We also examined the effect of overexpressing individual TRIM proteins on BZLF1 expression. Over-expression of TRIM33 and TRIM24 in AGS-EBV cells decreased spontaneous expression of BZLF1, consistent with repression of this gene (Fig 7D). The effects of TRIM33 and TRIM24 overexpression on the BZLF1 promoter (Zp) were also examined using a Zp-luciferase reporter assay in AGS cells, and both were found to consistently decrease the activity of Zp (Fig 7E). Together the results identify roles for TRIM33 and TRIM24 in repressing BZLF1 expression, likely through effects on Zp.

We also generated a TRIM33 SUMO mutant (SUMO^m^) in which the four lysines we detected as SUMO-modified by MS were changed to arginines (K769R, K774R, K776R and K793R). We verified that SUMO^m^ had reduced SUMOylation compared to WT TRIM33 by expressing the two proteins along with 6His-SUMO1 in 293T cells, then comparing their migration in SDS-PAGE both in cell lysates and after isolation of SUMO-modified proteins on metal chelating resin (Fig 7F). Both samples showed a loss of the slower migrating forms of TRIM33 in the SUMO^m^, indicative of less efficient SUMOylation, although SUMOylation was not completely abrogated. SUMO^m^ was then compared to WT TRIM33 for the ability to repress spontaneous BZLF1 expression in AGS-EBV cells (Fig 7G). Although some SUMO modification still appears to be present for the mutant, SUMO^m^ was less able to repress BZLF1 expression than WT TRIM33. This suggest that SUMO modifications contribute to this TRIM33 function, although other roles of these lysine residues cannot be ruled out.

### BZLF1 interacts with and disrupts the TRIM24/28/33 complex

**Fig 8:**
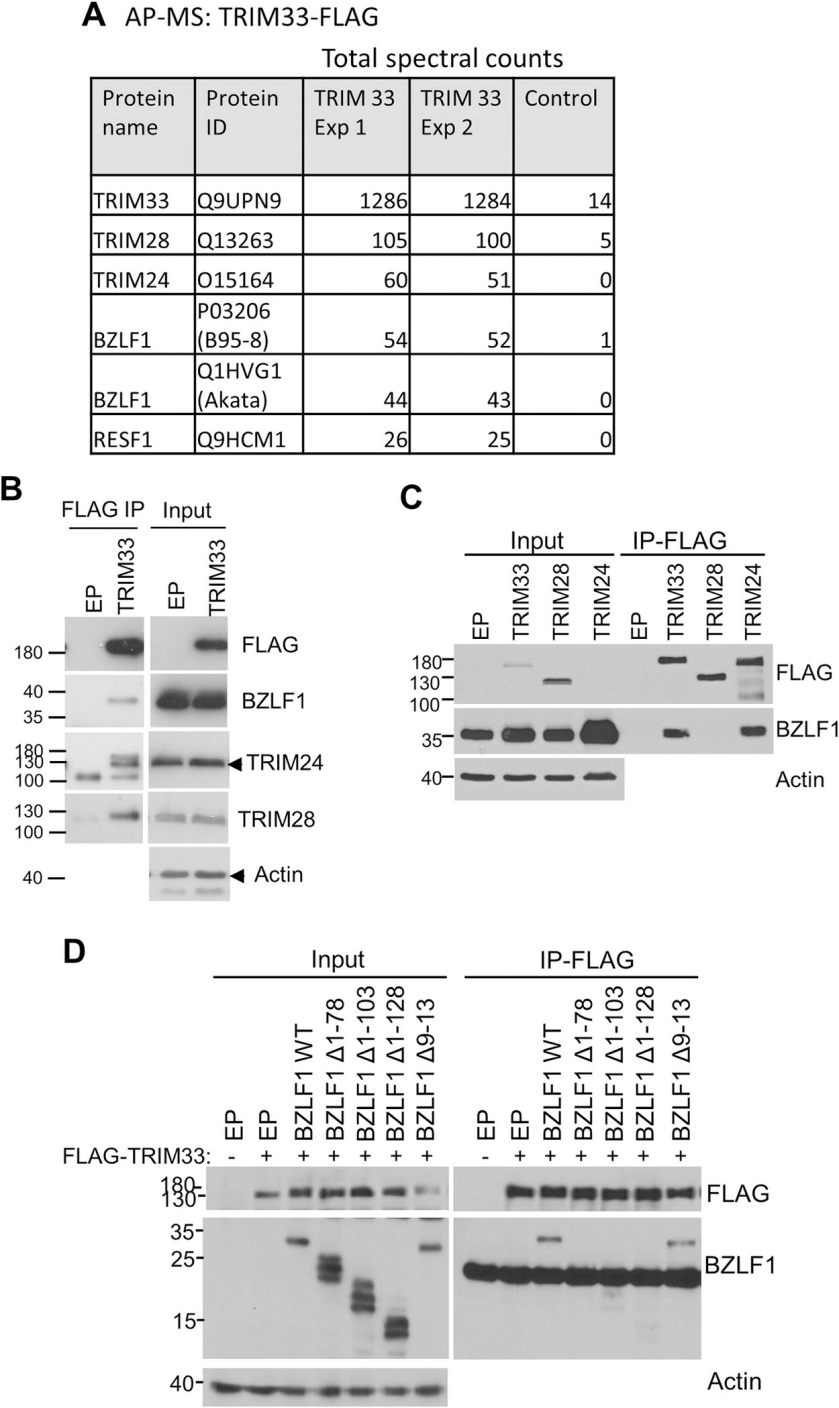
BZLF1 binds to TRIM24 and TRIM33. A. TRIM33-FLAG or empty FLAG (negative control) was expressed in AGS-EBV-Z cells followed by reactivation with dox for 24 hrs, and TRIM33 and associated proteins were isolated on anti-FLAG resin. Recovered proteins were identified by tandem MS. Total spectral counts are shown for the five most prevalent interactors seen in two independent experiments. B. TRIM33-FLAG was expressed in AGS-EBV followed by reactivation by sodium butyrate/TPA treatment and FLAG IPs. Western blots on IP samples and 10% of input samples are shown using the indicated antibodies. C. AGS cells were transfected with plasmids expressing BZLF1 and FLAG-tagged TRIM33, TRIM28, TRIM24 or empty FLAG plasmid (EP). 36 hours later, IPs were performed using FLAG antibody, followed by Western blotting with BZLF1 and FLAG antibodies. Input lysates were also probed with actin antibody. D. AGS cells were transfected with plasmids expressing FLAG-TRIM33 and BZLF1 WT or mutants (or empty plasmid) as indicated. IPs were performed using anti-FLAG M2 beads, followed by Western blotting as in C. The lower band in the BZLF1 blot of IP samples is from the IgG light chain.

To gain insight into the role of TRIM33 in EBV lytic infection, we expressed FLAG-tagged TRIM33 in AGS-EBV-Z cells, reactivated them to the lytic cycle with dox, then performed affinity purification of TRIM33-FLAG coupled to mass spectrometry. The top TRIM33 interactors from two independent experiments are shown in Fig 8A. As expected, prominent interactions were seen with TRIM28 and TRIM24, confirming that either dimeric or trimeric interactions occur between these proteins and TRIM33 in lytic EBV infection. The next two most highly recovered proteins were the two versions of BZLF1 expressed in these cells (which differ in sequence), one from the Akata virus that was reactivated and the other from the dox-inducible cassette (B95-8 EBV strain). These interactions were confirmed by immunoprecipitating TRIM33-FLAG from the EBV lytic infection lysates and Western blotting (Fig 8B).

To better understand the interaction of BZLF1 with the TRIM protein complexes, we tested the ability of BZLF1 to bind individual TRIM proteins. BZLF1 was co-expressed with FLAG-tagged TRIM24, TRIM28 or TRIM33 and FLAG IPs were performed (Fig 8C). BZLF1 was found to bind both TRIM24 and TRIM33 but not TRIM28, suggesting that the interaction of BZLF1 with TRIM protein dimers or trimers occurs through TRIM24 and TRIM33. Further co-IP experiments in which BZLF1 N-terminal mutants, with deletions in the transcriptional activation domain [45], were co-expressed with FLAG-tagged TRIM33 indicated that the interaction with TRIM33 occurred through this domain, and that deletion of amino acids 1–78 was sufficient to disrupt the TRIM33 interaction (Fig 8D). However, deletion of amino acids 9–13 did not disrupt the TRIM33 interaction, indicating that the SUMO-modified K12 is not important for this interaction.

**Fig 9:**
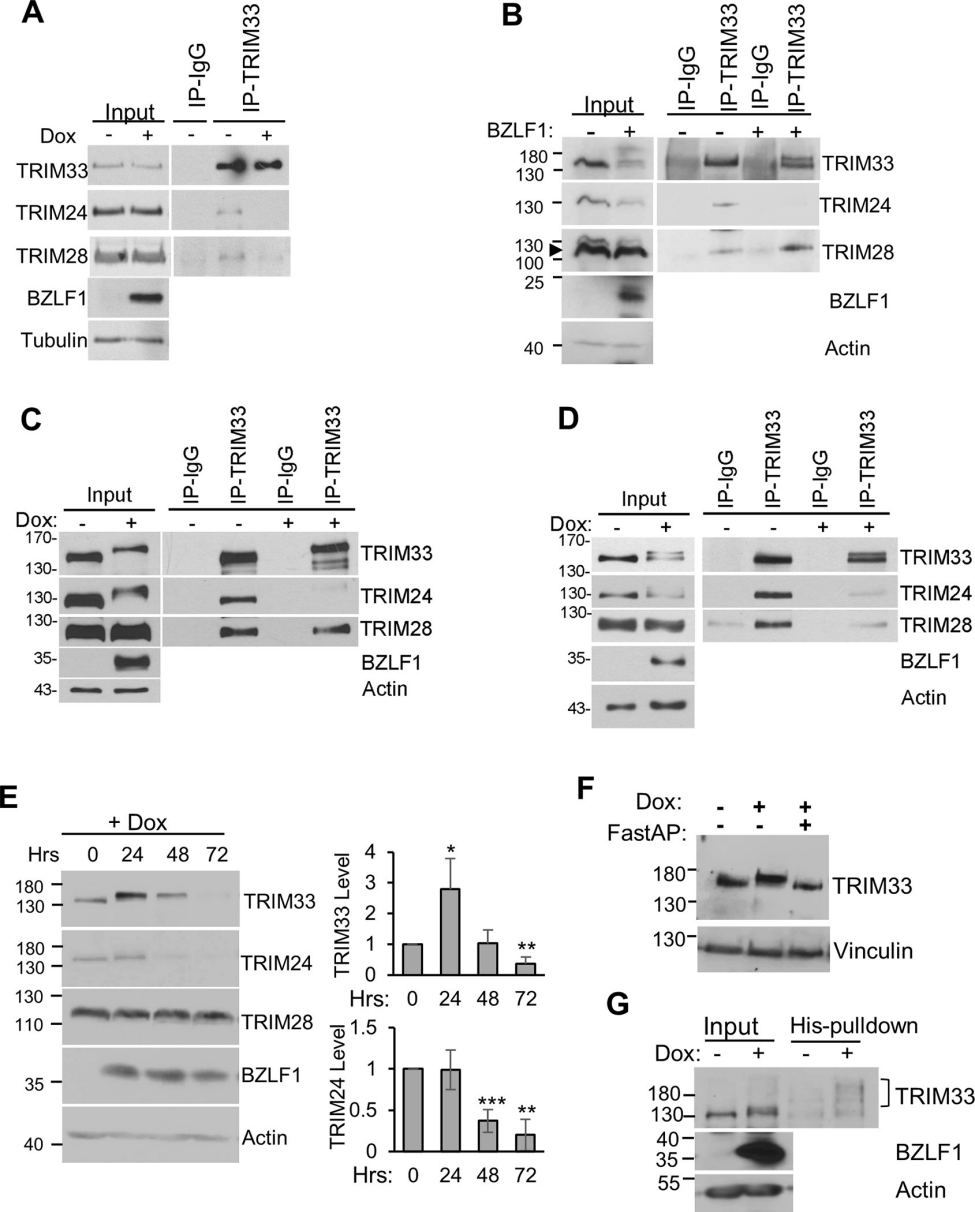
BZLF1 disrupts the TRIM24/TRIM33/TRIM28 complex. A. AGS-BZLF1 cells before (-) or 24 hrs after dox-induction of BZLF1 were used to perform IPs with TRIM33 or IgG control antibodies, followed by Western blotting with antibodies against TRIM24, TRIM28 and TRIM33. Input lysates were also probed with BZLF1 and tubulin antibodies. B. 293T cells were transfected with a BZLF1 expression plasmid (+) or empty plasmid (-), then IPs were performed with TRIM33 or IgG negative-control antibodies, followed by Western blot on inputs and IPs with antibodies against TRIM24, TRIM28 and TRIM33. Input lysates were also probed with BZLF1 and actin antibodies. C. NPC43-Z cells before (-) or 24 hrs after dox-induction of BZLF1 were used to perform IPs with TRIM33 or IgG control antibodies, followed by Western blotting with antibodies against TRIM24, TRIM28 and TRIM33. Input lysates were also probed with BZLF1 and actin antibodies. D Akata-Z cells before (-) or 24 hrs after dox-induction of BZLF1 were used to perform IPs with TRIM33 or IgG control antibodies, followed by Western blotting as in C. E. AGS-BZLF1 cells before (-) or the indicated hours after dox-induction of BZLF1 were analyzed by Western blotting with antibodies against TRIM33, TRIM28, TRIM24, BZLF1 and actin. TRIM bands were quantified from three independent experiments and average values with standard deviation were plotted. * = 0.01<P≤0.05, ** = 0.001< P ≤ 0.01, *** = 0.0001<P≤0.001. F. Whole cell lysates from AGS-BZLF1 cells with and without 24 hour dox induction were incubated with or without FastAP phosphatase, followed by Western blotting with TRIM33 and vinculin antibodies. G. AGS-BZLF1 cells were transfected with 6His-SUMO2 expression plasmid then treated with dox for 24 hrs or left untreated. 10% of cell lysates were analyzed as input samples. The remaining lysates were used to isolate His-SUMO-modified proteins on metal chelating resin. Samples were analyzed by Western blotting using TRIM33, BZLF1 and actin antibodies.

TRIM24/28/33 complex formation stabilizes TRIM24, enabling its antiviral activity [40,41,46]. The interaction of BZLF1 with TRIM24 and TRIM33 suggests a mechanism by which BZLF1 might disrupt and disable TRIM24/28/33 complexes. To test this possibility, we used EBV-negative AGS cells with a dox-inducible BZLF1. IPs of endogenous TRIM33 were performed before and after induction of BZLF1 expression and recovery of TRIM28 and TRIM24 was examined (Fig 9A). BZLF1 expression was found to decrease the recovery of TRIM24 (and to a lesser degree TRIM28) with TRIM33, consistent with disruption of the complex by BZLF1. Similar results were obtained when BZLF1 was expressed by transient transfection of 293T cells, where the presence of BZLF1 again reduced the recovery of TRIM24 with endogenous TRIM33 (Fig 9B). This higher level of BZLF1 expression was also found to decrease the input levels of TRIM33 and TRIM24, consistent with destabilization of the complex. We then examined whether the TRIM24/28/33 complex was similarly disrupted in the context of EBV lytic infection, using both NPC43-Z (Fig 9C) and Akata-Z (Fig 9D) cells. In both cases, immunoprecipitation of endogenous TRIM33 before and after EBV reactivation showed a major decrease in recovery of TRIM24 with TRIM33 after reactivation, with a smaller effect on recovery of TRIM28. Together the results indicate that both lytic reactivation and BZLF1 expression on its own consistently disrupts the interaction of TRIM24 with TRIM33, with a more variable effect on the TRIM33-TRIM28 interaction.

**Fig 10:**
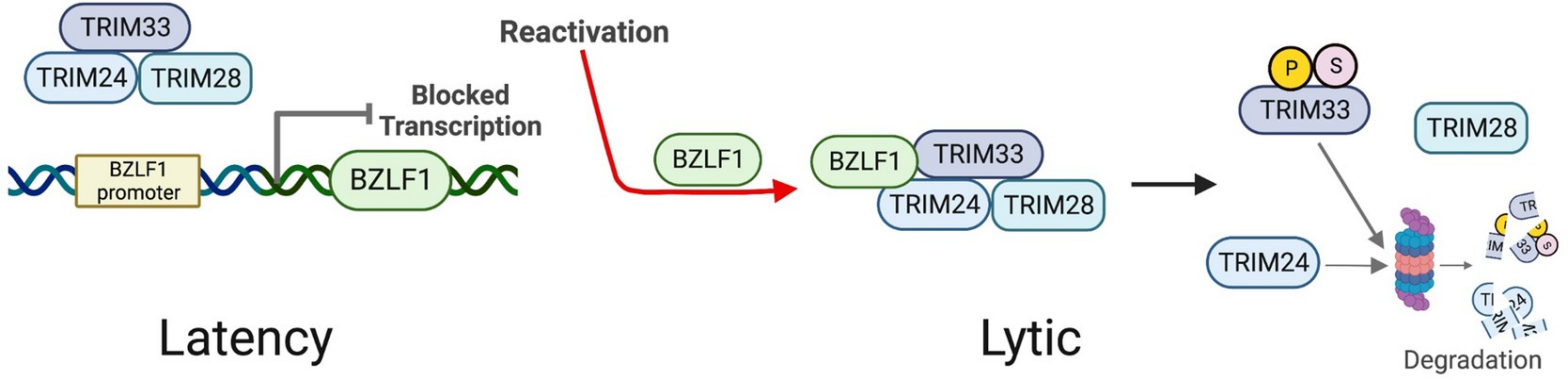
Model of the repression of the BZLF1 promoter by TRIM24/TRIM28/TRIM33 complexes in latent infection and their disruption by BZLF1 in lytic infection. The interaction of BZLF1 with TRIM24 and TRIM33 and/or the disruption of TRIM24/TRIM28/TRIM33 complexes by BZLF1 leads to degradation of TRIM24, and phosphorylation and SUMOylation of TRIM33 followed later by its degradation. Created with BioRender.com.

If BZLF1 disrupts the interaction of TRIM24 with TRIM33, then we would expect BZLF1 to destabilize TRIM24. We investigated this further by following the TRIM protein levels with time after BZLF1 expression in the dox-inducible AGS cells (Fig 9E). While TRIM28 levels remained stable, TRIM24 levels greatly decreased at 48 and 72 hours after BZLF1 induction. In addition, BZLF1 expression resulted in a slight mobility shift of TRIM33 at 24 hours post-induction (similar to the phospho-shift of TRIM33 seen in EBV lytic infection) and an initial increase in TRIM33 levels followed by a loss of TRIM33 at later time points. Treatment of the lysate from the 24 hour time point with phosphatase shifted TRIM33 back to its original position (Fig 9F), indicating that BZLF1 expression alone was sufficient to induce phosphorylation of TRIM33. We also examined whether BZLF1 expression induced SUMOylation of TRIM33, by expressing 6His-SUMO2 in the dox-inducible BZLF1 cells then comparing recovery of 6His-purified proteins before and 24 hours after induction of BZLF1. Western blots for TRIM33 revealed an increase in SUMO-modified TRIM33 after BZLF1 expression, indicating that BZLF1 was sufficient to induce TRIM33 SUMOylation and further demonstrating the co-regulation of TRIM33 phosphorylation and SUMOylation (Fig 9G). Together the results suggest that the interaction of BZLF1 with TRIM33 and TRIM24 in the context of lytic infection triggers phosphorylation and SUMOylation of TRIM33, as well as disruption of the TRIM complex, resulting in destabilization of TRIM24 and TRIM33 (Fig 10).

## Discussion

Several studies suggest a dynamic regulation of host SUMOylation during EBV infection [23–25,27,28,38]. In this study, we provide a comprehensive proteomic analysis of host SUMO1 and SUMO2 modified proteins during EBV latent and lytic infection, identifying 1726 modification sites in 828 proteins. Prominent findings include that lytic reactivation results in induction of the highly SUMO-modified stem cell factor SALL4, increased SUMOylation of SUMO proteins themselves, and altered SUMOylation of NuRD complex, nuclear pore and actin binding proteins, suggesting alterations in their functions. In addition, significant changes were identified either to the SUMOylation status or the cellular abundance in all three components of the TRIM24/TRIM28/TRIM33 complex. TRIM33 is one of the earliest responders to EBV lytic infection, which triggers a large increase in its phosphorylation and SUMOylation, followed by the proteasomal degradation of TRIM24. We showed that both TRIM24 and TRIM33 repress expression of the EBV BZLF1 lytic switch gene and identified a mechanism by which BZLF1 disrupts the interactions and stabilities of these proteins to overcome this repression.

Our proteomic study also identified seven SUMO-modified EBV proteins, as well as the sites of these modifications, including two proteins (BORF2 and BALF2) not previously reported to be SUMOylated. For BMRF1 and BZLF1, phosphorylation sites were also identified within the SUMO-modified peptides. Consistent with previous reports, we found the immediate early transcription factors, BRLF1 and BZLF1, were both SUMO-modified. The SUMO-modified site in BRLF1 that we identified (K530) is different from those previously reported by Chang et al., 2004 [37] but consistent with mutational analysis of potential SUMO sites reported by Heilmann et al., 2010 [39]. We identified two SUMO-modified sites in BZLF1 (K12 and K161), one of which (K12) was previously shown to be important for transactivation by BZLF1, in that mutation of this site enhances transcriptional activity [47]. This suggests that one reason that inhibiting SUMOylation increases EBV lytic infection might be due to an increase in transcriptional activation by BZLF1.

TRIM proteins regulate antiviral signaling pathways and have roles in intrinsic resistance to DNA and RNA virus infections [48,49]. PML/TRIM19, alone or in conjunction with other proteins in PML nuclear bodies, is a well-known restriction factor for several viruses [50–52]. TRIM24, TRIM28 and TRIM33 belong to a subfamily of TRIM proteins (TIF1 family) that associate with chromatin through their PHD-bromo domains, regulate gene transcription and have E3 ubiquitin ligase activity [53]. All three also have roles in regulating viral infections. TRIM28 contributes to epigenetic silencing of endogenous and exogenous retroviruses by recruiting the H3K9 lysine methyltransferase SETDB1/ESET to retrovirus sequences, although roles in activating HIV-1 gene expression have also been reported [54,55]. In addition, TRIM28 has been shown to regulate lytic reactivation in CMV, EBV and KSHV by repressing viral gene expression [26,44,56–58]. Similarly to TRIM28, TRIM24 and TRIM33 have been found to repress expression of VL30 endogenous retrovirus (ERV) elements in mouse hepatocytes [46]. TRIM24 has also been shown to be part of the antiviral response to VSV infection, in which TRIM24 translocates to mitochondria where it binds TRAF3 and mediates its K63-linked ubiquitination, resulting in activation of TRAF3 signaling [59]. TRIM33 has been reported to restrict infection of multiple viruses by a variety of mechanisms. TRIM33 inhibits HIV-1 infection by ubiquitinating and inducing the proteasomal-mediated degradation of the viral integrase, thus preventing viral cDNA integration into the cellular genome [60]. TRIM33 was also found to repress the long terminal repeat (LTR) of the MMERVK10C retrotransposon through ubiquitination and degradation of the host A-MYB protein that activates transcription from these elements [61]. In addition, TRIM33 limits adenovirus gene expression and its degradation is caused by a variety of adenoviruses, suggesting that it is a restriction factor for adenovirus infection [62]. Here we show that TRIM24 and TRIM33 repress expression of the EBV lytic switch protein, BZLF1, thereby inhibiting EBV lytic reactivation.

In addition to the structural and functional relationships among TRIM24, TRIM28 and TRIM33, these proteins have also been shown to physically interact to form dimeric and trimeric complexes, raising the possibility that some of their identified roles might be due to the action of the complex [40,41,46,63]. TRIM24 is a very labile protein that is protected from ubiquitylation and proteasomal degradation by interactions with these TRIM proteins as well as with p53 [40,42,64]. Therefore, the integrity of TRIM24/TRIM33, TRIM24/TRIM28 or TRIM24/TRIM28/TRIM33 complexes may be essential in preserving the antiviral function of TRIM24, making disruption of the complexes an attractive pro-viral target. Since TRIM24 is an E3 ligase, it can undergo auto-ubiquitylation and has also been found to be ubiquitylated by cellular (speckle-type POZ protein) and viral (adenovirus E4orf3) ubiquitin ligases [42,62,64].

SUMO modification of TRIM33 has been previously reported and shown to be part of the mechanism by which TRIM33 represses TGFβ signaling [65]. In that study, SUMOylation of TRIM33 was abrogated by mutating four lysines in the central region of TRIM33. We also found this region of TRIM33 to be consistently SUMO-modified at four lysines (K769, K774, K776 and K793) in response to EBV reactivation, although only two, K776 and K793, were in common with the previously mapped sites. In addition to SUMO modifications, we found phosphorylation of TRIM33 at three residues (T781, S787 and S789). Additional phosphorylation sites have been reported in TRIM33 [66,67], but these would not have been detected in our study, as our proteomic survey of the TRIM33 sequence was restricted to the serine and threonine resides found in peptides also containing a Gly-Gly-K derived from SUMO. EBV-induced phosphorylation of TRIM33 is so extensive that 100% of the protein is seen to shift in SDS-PAGE gels in early lytic infection in AGS and NPC43 (Fig 6B and 6D), with ~50% shifting in Akata cells (Fig 6E, top panel). Interestingly, a similar shift in mobility of TRIM33 is seen in response to adenovirus infection, which also results in TRIM33 SUMOylation and proteasomal degradation [62,68,69], although the nature of this shift was not determined. The adenovirus SUMO ligase, E4-ORF3, appears to be responsible for this SUMOylation, and its deletion also results in loss of the TRIM33 mobility shift suggesting that, as in EBV infection, these TRIM33 modifications are linked [62,68,69].

Viruses typically evolve mechanisms to overcome antiviral restriction, and here we have discovered a novel role for BZLF1 in disabling such a host defense. Together our data support the model shown in Fig 10, in which complexes involving TRIM24, TRIM28 and TRIM33 initially restrict BZLF1 expression, but are inactivated by BZLF1 once it is expressed. BZLF1 interacts with TRIM24 and TRIM33 and interferes with the interaction of these proteins, resulting in destabilization and proteasomal degradation of TRIM24. BZLF1 expressed in the absence of EBV infection is also sufficient to induce phosphorylation and SUMOylation of TRIM33, which may be a result of the disruption of TRIM24/TRIM28/TRIM33 complexes and/or a conformational change in TRIM33 induced by interaction with BZLF1, making the TRIM33 sites accessible to endogenous kinases and SUMOylation machinery. While TRIM33 is not as labile as TRIM24, the disruption of its interactions with TRIM24 and/or its phospho- and SUMO-modifications induced by BZLF1 resulted in TRIM33 destabilization.

In summary, we identified TRIM24 and TRIM33 as restriction factors for EBV lytic infection, and shown that they are dramatically altered in response to EBV reactivation as a result of the action of the EBV BZLF1 lytic switch protein, that disables this host antiviral response.

## Materials and methods

### Cell lines

AGS gastric carcinoma cells, EBV-positive AGS cells (AGS-EBV) [70], AGS-EBV cells with Tet-inducible BZLF1 (AGS-EBV-Z) [32], and NPC43 EBV-positive nasopharyngeal carcinoma cells (a gift from Sai Wah Tsao) [43] were all previously described and were cultured in RPMI (GIBCO) with 10% FBS. The EBV-positive HONE-Akata cell line, originally derived from nasopharyngeal carcinoma cells [71], has been demonstrated to contain sequences derived from HeLa cells as well as HPV18 [72,73]. These cells were cultured in α-MEM (GIBCO) with 10% FBS. HEK293T cells were cultured in DMEM (GIBCO) with 10% FBS. All media was supplemented with 100 U/ml penicillin and 100 μg/ml streptomycin (Gibco). Medium for NPC43 cells also contained 4 μM ROCK inhibitor (Enzo Y-27632), unless otherwise indicated. Media for AGS-EBV and HONE-Akata also contained 80 μg/ml geneticin (G418). Medium for AGS-EBV-Z was supplemented with 2 μg/ml puromycin for two passages before setting up the experiments.

AGS cells with Tet-inducible BZLF1 (AGS-BZLF1) were generated by transfecting AGS cells in a 6-well plate with 1 μg of Ssp I-linearized pTRIPZ containing a BZLF1 expression cassette (pTRIPZ-BZLF1) [74]. Twenty-four hours post-transfection, puromycin was added to 2 μg/ml for 2 days. Cells were moved to a 15-cm dish containing RPMI medium supplemented with 2 μg/ml of puromycin. Two weeks post-transfection, colonies were picked with trypsin-soaked Whatman paper and propagated as for AGS-EBV cells. The Akata EBV-positive Burkitt’s lymphoma cell line [75] and NPC43 cells were both engineered to contain an integrated Tet-inducible BZLF1 (Akata-Z and NPC43-Z, respectively). These were generated by transducing Akata and NPC43 cells, grown in RPMI+10% FBS in a 6-well plate, with lentivirus made from pTRIPZ containing a BZLF1 expression cassette (pTRIPZ-BZLF1; [74]). Twenty-four hours post-transduction, puromycin was added to 2 μg/ml to select for cells containing the cassette. AGS cells containing the empty TRIPZ plasmid was generated by transducing AGS cells with lentivirus made from the TRIPZ plasmid and grown under puromycin selection as above. To generate AGS cells with dox-inducible GFP (AGS-GFP), a blasticidin expression cassette was inserted in the Kpn I site in TRIPZ-GFP [74]. Lentivirus generated from this construct was used to transduce AGS cells, which were grown in 10 μg/ml blasticidin to select for cells containing the construct.

### Plasmids

paPX1-SUMO1-KGG-mCherry and paPX1-SUMO2-KGG-mCherry PiggyBac plasmids expressing 6His-SUMO1-T95K-mCherry and 6His-SUMO2-T90K-mCherry, respectively, were described previously [14]. pUC19 encoding the Supper Piggy Bac transposase from Supper Piggy Bac transposase expression plasmid (SBI Biosciences) was described previously [76]. pcDNA-His-SUMO1 [77] and pcDNA-His-SUMO2 [78] were described previously. pCMX-FLAG-TRIM28 (124960) and pCMX-FLAG-TRIM24 (28138) were purchased from Addgene. TRIM33 in pSG5 (a gift from Ruth Rimokh) was amplified by PCR using oligos forward KpnI 5’–ACG TGG TAC CGC CGC CAT GGC GGA AAA CAA A—3’ and reverse BamHI 5’–ACG TGG ATC CTT GCT TTA TAT GTA CTG GTC TCT CA– 3’ and inserted between the Kpn I and Bam HI sites in pCMV3FC [79] to generate TRIM33 with C-terminal FLAG. The TRIM33 SUMO mutant containing the substitutions K769R, K774R, K776R and K793R was generated using STRING (Invitrogen) with Eco NI and Bam HI sites to subclone between the Eco NI and Bam HI in pCMV3FC-TRIM33. pLentiCRISPRv2-LoxP [80] was a gift from Reuben Harris. pPL17 expressing BZLF1 [81] was a gift from Dong-Yan Jin. The BZLF1 promoter (Zp) firefly luciferase reporter plasmid (pZp-luc) was provided by Takayuki Murata [82] and the renilla pRL-promoterless control plasmid was from Promega.

### Generation of 6His-SUMO1 and 6His-SUMO2 cells

AGS-EBV-Z-6His-SUMO1-T95K and AGS-EBV-Z-6His-SUMO2-T90K cells were generated as follows: AGS-EBV-Z cells in 10 cm dishes were transfected with 4 μg of paPX1-SUMO1-KGG-mCherry or paPX1-SUMO2-KGG-mCherry along with 1 μg of Supper PiggyBac transposase expression vector. Cells were passaged twice as described above for AGS-EBV-Z cells and mCherry positive cells were selected by fluorescence-activated cell sorting (FACS) using a FACSAriaII cytometer. Cells were expanded and confirmed to express 6His-SUMO1- or 6His-SUMO2-mCherry by fluorescence microscopy and Western blotting.

### Cell lysates for mass spectrometry experiments

AGS-EBV-Z-6His-SUMO1 and AGS-EBV-Z-6His-SUMO2 cells at 80–90% confluency in 15 cm dishes were left untreated or treated with doxycycline for 12 or 24 hours to induce the lytic cycle. Three replicates of each condition were prepared. Cells from one dish were lysed in 9M urea buffer, sonicated and clarified by centrifugation. 50 μg of these lysates were subjected to SDS-PAGE (6% to 15%), transferred to nitrocellulose and analyzed by Western blotting with the indicated antibodies. Cells from 20 dishes for each condition were used to prepare samples for proteomic analyses. These cells were washed twice with cold Dulbecco’s phosphate-buffered saline (DPBS) and lysed in 6 ml of cell lysis buffer [6 M guanidinium-HCl, 100 mM sodium phosphate buffer (pH 8.0), 10 mM Tris-HCl (pH 8.0), 20 mM imidazole and 5 mM β-mercaptoethanol] per gram of cell pellet. These lysates were use both for total protein analysis and for isolation of SUMO-conjugated peptides as described below.

### Mass spectrometry analysis of total protein

Crude cell extracts were generated by TCA precipitation of proteins from denaturing lysates described above, and then solubilized by addition of 1.2x NuPAGE sample buffer with reducing agent. Approximately 25μg protein was fractionated by SDS-PAGE (NuPage 10% polyacrylamide, Bis-Tris with MOPS buffer; Invitrogen) and stained with Coomassie blue. Each lane was sliced into 5 sections and peptides extracted by tryptic digestion [83] including alkylation with chloroacetamide. Final peptides were resuspended in 0.1% TFA 0.5% acetic acid and peptide samples analysed by LC-MS/MS. This was performed using a Q Exactive mass spectrometer (Thermo Scientific) coupled to an EASY-nLC 1000 liquid chromatography system (Thermo Scientific), using an EASY-Spray ion source (Thermo Scientific) running a 75 μm x 500 mm EASY-Spray column at 45°C. A 150 minute elution gradient with a top 10 data-dependent method was applied. Full scan spectra (m/z 300–1800) were acquired with resolution R = 70,000 at m/z 200 (after accumulation to a target value of 1,000,000 ions with maximum injection time of 20 ms). The 10 most intense ions were fragmented by HCD and measured with a resolution of R = 17,500 at m/z 200 (target value of 500,000 ions and maximum injection time of 60 ms) and intensity threshold of 2.1x10^4^. Peptide match was set to ‘preferred’, a 40 second dynamic exclusion list was applied and ions were ignored if they had unassigned charge state 1, 8 or >8. Data analysis used MaxQuant version 1.6.1.0 [84]. Default settings were used with a few exceptions. A database of 84 EBV proteins along with the uniport human proteome database (downloaded 19/4/2019–73920 entries) was used as search space. Digestion was set to Trypsin/P (ignoring lysines and arginines N-terminal to prolines) with a maximum of 3 missed cleavages. Match between runs was enabled, which matched identified peaks from different samples among slices from the same position in the gel as well as one slice higher or lower. Protein, peptide and modification level FDR was set to 1%. Label-free quantification (LFQ) was enabled with normalization skipped. Manual LFQ normalization was done post analysis by first calculating the relative LFQ intensity (ratio of LFQ intensity in a slice compared to average LFQ intensity across all equivalent slices) for each protein in each slice. This was done only for proteins with LFQ intensities reported in all 18 equivalent slices (all replicates for all conditions). The median relative intensity of all proteins in a slice was used to normalize all protein LFQ values for that slice. The final protein LFQ intensity per lane (and therefore sample) was calculated by the sum of normalized LFQ values for that protein in all five slices. Downstream data processing used Perseus v1.6.1.1 [85]. Proteins were only carried forward if an LFQ intensity was reported in all three replicates of at least one condition. Zero intensity values were replaced from log_2_ transformed data (0.3 width, 1.8 downshift) and outliers were defined by 5% FDR from Student’s t-test using an S0 value of 0.1. MS data were also analysed a second time with identical settings except for including variable modification for Phospho (STY) with 1% FDR filtering at the site level.

### Mass spectrometry analysis of SUMO-modified peptides

SUMO modified peptides were prepared from each cell lysates as described previously [14,86]. In brief, NiNTA chromatographic enrichment of 6His-SUMO conjugates was undertaken from 20 mg crude cell lysate and elutions from the NiNTA columns were digested consecutively with LysC then GluC prior to GlyGly-K immunoprecipitation. The final enriched fractions of LysC and LysC+GluC GG-K peptides were resuspended in a volume of 20 μl for two proteomic analyses. Firstly, 2 μl was fractionated over a 90 minute gradient with the 4 most intense ions fragmented with a resolution of R = 35000 at m/z 200 (target value of 1,000,000 ions and maximum injection time of 300 ms) and intensity threshold of 2.1x10^4^. Secondly, 15μl of sample was fractionated over a 150 minute gradient and the 2 most intense ions fragmented with a resolution of R = 70000 at m/z 200 (target value of 1,000,000 ions and maximum injection time of 800 ms) and intensity threshold of 2.1x10^4^. Both MS runs were processed together in MaxQuant as described above with each sample given a separate experimental identifier so peptide intensity values were reported for every run on the MS. The same sequence databases as above were used although digestion with LysC (3 missed cleavages), or LysC+GluC_D/E (considering cleavage after D or E and 8 missed cleavages) were considered. GlyGly (K) and phospho (STY) modifications were selected and maximum peptide mass was increased to 7000. 1% FDR filtering was applied at protein, peptide and site levels. Matching between runs was allowed but only for peptide samples digested by the same protease and fractionated with the same LC elution gradient. Manual normalization followed a similar method as described above where ‘equivalent’ peptide samples (ie. those digested by the same protease and with the same peptide elution gradient) from different replicates were compared with one another. For each peptide common to all equivalent peptide samples the intensity relative to the average intensity for that peptide across all samples was calculated. The median of that relative intensity in a sample (for all peptides also found in equivalent samples) was used to normalize all peptide intensities for that sample. The final peptide intensity per experimental replicate was calculated by the sum of all normalized intensities in samples derived from that replicate no matter the enzyme or HPLC gradient. Importantly, peptide samples derived from SUMO1 and SUMO2 cells were considered equivalent for normalization purposes, which assumes largely similar abundances of peptides across cell types. Zero intensity values were replaced as described above, and outliers also defined by the same 5% FDR criteria.

The mass spectrometry proteomics data have been deposited to the ProteomeXchange Consortium via the PRIDE [87] partner repository. Project accession: PXD034631.

### Bioinformatic analysis of the SUMO site proteomics

726 proteins identified with at least one SUMO1 or SUMO2 modification site were uploaded to STRING [88] for network and functional group enrichment analysis. Only proteins associated by a minimum STRING interaction score of 0.7 (high confidence) were included in the final network. Disconnected nodes were removed. Selected groups of functionally related proteins were resubmitted to STRING to create smaller sub-networks. All networks were visualised in Cytoscape v 3.7.2 [89] allowing the graphical display of numbers of sites identified total GG-K peptide intensity and rough proxy for the rate of change in total SUMOylation during EBV reactivation.

### EBV reactivation and lytic cycle analysis

The lytic cycle in AGS-EBV-Z and AGS-EBV-Z derived cells was induced by the addition of 2 μg/ml doxycycline to the medium. To induce the EBV lytic cycle in AGS-EBV, HONE-Akata, and NPC43 cell lines, cells were treated with 20 ng/ml 12-O-tetradecanoylphorbol-13-acetate (TPA) and 3 mM sodium butyrate (NaB) for the indicated times. For NPC43 cells, the ROCK inhibitor was removed from the medium for 5 days prior to this treatment. In experiments involving ML792, cells were treated with 0.5 μM ML792 (or equivalent volume of DMSO as a negative control) for 24 hours prior to TPA/NaB addition. In all cases, cells were lysed in in 9M urea buffer (9M urea, 10 mM Tris pH 6.8) and lysates were sonicated and clarified by centrifugation. 50 μg of clarified lysate (120 μg of for NPC43) was analyzed by Western blotting with the indicated antibodies. To determine the percentage of cells in the lytic cycle, AGS-EBV-Z derived cells were fixed and stained with anti-BZLF1 antibody 16 hours after doxycycline addition and analysed by fluorescence microscopy as described below.

### Validation of changes in SUMOylation of cellular and viral proteins during EBV lytic infection

AGS-EBV-Z-6His-SUMO1 and AGS-EBV-Z-6His-SUMO2 cells at 80–90% confluency were left untreated or treated with 2 μg/ml doxycycline and harvested at 0 (uninduced), 12 and 24 hours post-induction. 10% of cells were lysed in 9M urea buffer, sonicated and clarified by centrifugation to provide the input sample. 90% of cells were lysed in buffer G (6 M guanidine hydrochloride, 10 mM Tris, 100 mM sodium phosphate, pH 8.0) and 6His-tagged SUMO conjugates were purified as previously described [27]. Inputs and purified fractions were analyzed by Western blotting with specific antibodies for the protein in question. Further validation of TRIM33 SUMOylation involved comparing AGS-EBV-Z, AGS-EBV-Z-6His-SUMO1 and AGS-EBV-Z-6His-SUMO2 cells with and without treatment with 0.5 μM ML792 for 16 hours, followed by doxycycline induction for 24 hours. Cells were then lysed in 9M urea buffer. Lysates were sonicated, clarified by centrifugation and 50 μg of each was analysed by Western blotting with the indicated antibodies.

For experiments in NPC43-Z and Akata-Z cells, cells in 6 well plates were treated with 2μg/ml doxycycline for 24 or 48 hours to induce BZLF1 expression or left untreated. Cell lysates were generated as above and 30 μg of clarified lysates were analyzed by Western blotting with the indicated antibodies. In some experiments, NPC43-Z and Akata-Z cells were treated with 0.5 μM ML792 or DMSO (negative control) with or without 2μg/mL doxycycline for 12 (NPC43-Z) or 24 (Akata-Z) hours, prior to lysis and Western blotting of 50 μg (NPC43-Z) or 30 μg (Akata-Z) clarified lysate.

### Proteasomal degradation of TRIM24

AGS-EBV-Z or NPC43-Z cells in 6 well plates were treated with or without 2 μg/ml doxycycline along with 10 μM MG132 (in DMSO) or DMSO alone. Twelve hours later, cells were lysed in 9M urea buffer and clarified lysates were analysed by Western blotting.

### TRIM33 phosphorylation experiments

AGS-EBV-Z cells were left untreated or treated with 2 μg/ml doxycycline or doxycycline along with 0.5 μM ML792 and harvested after 12 hrs. Cells were lysed in 9M urea buffer and 10 μg of lysate was incubated with or without 10 U of FastAP Thermosensitive Alkaline Phosphatase (EF0651, ThermoFisher Scientific) in a 30 μl reaction at 37°C for 15 min. Lysates were then analyzed by Western blotting with the indicated antibodies. AGS-BZLF1 cells were treated with 2 μg/ml doxycycline and harvested after 24 hrs. Cells were lysed and incubated with FastAP as above except that 25 μg of lysate was used.

### SUMO1 silencing

AGS-EBV cells were plated in a 6-well dish at 20% confluency and transfected with either 40 pmoles of siSUMO1 (Santa Cruz) or Allstars control siRNA (Qiagen) using Lipofectamine RNAiMAX (Invitrogen) according to the manufacturer’s instructions. The transfection was repeated after 24 and 48 hours. 24 hours later, cells were lysed in 9M urea buffer for Western blot analysis or fixed and stained with anti-BZLF1 for fluorescence microscopy analysis. Lysates were sonicated and clarified by centrifugation. 80 μg of clarified lysate were analyzed by Western blotting with SUMO1, BZLF1 and actin antibodies. The percentage of reactivated cells was determined by performing immunofluorescence microscopy for BZLF1 (as described below), and counting ~300 cells for each sample in three independent experiments.

### Western blotting and antibodies

Inputs and protein fractions were separated by SDS-PAGE (6% to 15% depending on the experiment) and transferred to nitrocellulose. Membranes were blocked and incubated with primary and secondary antibodies as described previously [27]. Antibodies against BZLF1 (sc53904), BORF2 (sc56979), actin (sc47778), vinculin (sc-25336), SUMO1 (sc-9060), TRIM24 (sc271266), TRIM28 (sc136102), RanBP2 (sc74518), RanGAP1 (sc28322), Nup50 (sc398993), hnRNPK (sc28380), GATAD2B (sc101052), sp100 (sc25568), BRD7 (sc376180), and Sall4 (sc101147) were from Santa Cruz. Antibodies against FLAG (A190102A), HDAC2 (A300705A), RbBP4 (A301206A), and RbBP7 (A300959A) were from Bethyl. Antibodies against CHD4 (ab70469), MTA2 (ab8106), GATAD2A (ab87663) were from Abcam. Antibodies against BALF2 (OT13N) and Vcap18 (OT15E) were gifts from Jaap Middeldorp. Additional antibodies were anti-BMRF1 (MAB8186; Millipore), anti-FLAG M2 (F1084; Sigma-Aldrich), anti-FLAG (740001, Invitrogen), anti-TRIM33 (PA5-82152; Invitrogen), anti-ZNF451 (25228-1-AP; Proteintech). Secondary antibodies goat anti-mouse horseradish peroxidase (HRP) (sc-2005; Santa Cruz), donkey anti-goat HRP (SAB3700285; Sigma), goat anti-rabbit HRP (SAB3700878; Sigma), and goat anti-rat HRP (24555; ThermoFisher Scientific) were used at 1:5000 dilution. Signals were detected by enhanced chemiluminescence (Santa Cruz sc-2048 or Amersham ECL Prime RPN2232).

### Immunofluorescence microscopy

Cells were plated in 6-well dishes containing coverslips and treated as described in each experiment. Cells were fixed, permeabilized and blocked as described previously [74]. To detect lytic reactivation, coverslips were incubated with anti-BZLF1 (Santa Cruz, sc-53904, 1:200) for 1 hour at room temperature, washed with PBS, and incubated with anti-Mouse Alexa Fluor 488 at a 1:700 dilution. Cover slips were mounted onto slides and images were acquired as described previously [74].

### Generation of TRIM33 and TRIM24 knockout cells

Disruption of TRIM33 and TRIM24 genes was performed by the CRISPR-Cas9 system using CRISPR guide RNA sequences reported in Sanjana et al. 2014 [90]. A CRISPR guide RNA sequence targeting the Adeno-Associated Virus Integration Site 1 (AAVS1) was used as a negative control as previously described [91]. Guide oligonucleotides (S3 Table) were cloned into the Bsm BI site in pLentiCRISPRv2-LoxP. Lentiviruses were generated in HEK293T cells by standard methods and used to transduce AGS-EBV, HONE-Akata, and NPC43 cells. After 48 hours, cells were subjected to antibiotic selection with 2 μg/ml (AGS-EBV and HONE-Akata) or 1 μg/ml (NPC43) puromycin for 2 weeks to generate pools of TRIM33 knockout (KO), TRIM24 KO or control KO cells. Cells were then analyzed by Western blotting with TRIM33 or TRIM24 antibodies for successful gene disruption.

### Spontaneous EBV reactivation in TRIM33 and TRIM24 knockout cells

TRIM33 KO, TRIM24 KO and control KO pools of AGS-EBV, HONE-Akata and NPC43 cells were lysed in 9 M urea buffer, and the lysates were sonicated and clarified by centrifugation. NPC43 cells were cultured without ROCK inhibitor for 5 days prior to lysis. Clarified lysates (100 μg for AGS-EBV, 120 μg for HONE-Akata and 150 μg for NPC43) were analysed by 10% SDS-PAGE and Western blotting with the indicated antibodies.

### Affinity purification-mass spectrometry (AP-MS) of TRIM33 in EBV infection

AGS-EBV-Z cells in three 15-cm dishes were transfected with 10 μg of pCMV3FC-TRIM33 or pCMV3FC empty plasmid (negative control) using 30 μl of linear polyethylenimine (PEI). 24 hours post-transfection, cells were treated with doxycycline to induce the lytic cycle. Cells were harvested 24-hours post-doxycycline treatment and lysed in 4 volumes of RIPA buffer (50 mM Tris-HCl (pH 8.0), 300 mM NaCl, 0.1% sodium deoxycholate, 0.5% NP40 substitute, 2 mM EDTA) containing protease inhibitor cocktail (P8340, Sigma-Aldrich), sonicated and clarified by centrifugation. 6 mg of clarified lysate was incubated at a concentration of 8 μg/μl with 25 μl of anti-FLAG M2 magnetic beads (Sigma-Aldrich) for 4 hours at 4°C. Resin was washed three times with RIPA buffer and proteins were eluted and trypsinized as described in Cao et al. [92] Mass spectrometry and analysis was performed as previously described [32].

### TRIM24/TRIM28/TRIM33 and BZLF1 co-immunoprecipitation

AGS-EBV cells were transfected with pCMV3FC expressing FLAG-TRIM33 or pCMV3FC empty vector, then, 24 hours later, treated with 3 mM sodium butyrate and 20 ng/ml TPA to induce lytic reactivation. 24 hours later, cells were lysed in 4 volumes of modified RIPA buffer (50 mM Tris-HCl (pH 8.0), 300 mM NaCl, 0.1% sodium deoxycholate, 0.5% NP40 substitute, 2 mM EDTA) containing protease inhibitor cocktail (P8340, Sigma-Aldrich), sonicated and clarified by centrifugation. 500 μg of lysate was incubated with 2.5 μl of anti-FLAG M2 magnetic beads (M8823, Sigma-Aldrich) overnight at 4°C. The beads were then washed 4 times in 1 ml RIPA buffer and proteins were eluted in 2x SDS loading buffer. The eluates and 70 μg of clarified lysates (inputs) were analyzed by Western blotting.

To assess BZLF1 interaction with individual TRIM proteins, AGS cells were co-transfected with pPL17-BZLF1 and plasmids expressing FLAG-tagged TRIM24, TRIM28 or TRIM33 (or pCMV3FC empty plasmid). 36 hrs post-transfection, cells were lysed in 4 volumes of RIPA buffer (50 mM Tris-HCl (pH 8.0), 250 mM NaCl, 0.1% sodium deoxycholate, 0.5% NP40 substitute, 2 mM EDTA) containing protease inhibitor cocktail (P8340, Sigma-Aldrich), sonicated and clarified by centrifugation. 1 mg of clarified lysate was incubated at 4°C with agarose-conjugated FLAG antibody for 4 hours with mixing. Agarose beads were then washed 4 times with 1 ml RIPA buffer and proteins were eluted in 2x SDS loading buffer. The eluates and 50 μg of clarified lysates (inputs) were analyzed by Western blotting with antibodies against FLAG, BZLF1 and actin. The FLAG-TRIM33 co-IP experiments in AGS cells were also repeated with co-expression of WT BZLF1 or BZLF1 deletion mutants Δ1–78, Δ1–103, Δ1–128 and Δ9–13 (described in [45]) and FLAG IPs were performed 48 hrs post-transfection as above, but with 2.5 μl of anti-FLAG M2 magnetic beads (M8823, Sigma-Aldrich). Beads were then washed 4 times in 1 ml RIPA buffer and proteins were eluted in 2x SDS loading buffer. The eluates and 70 μg of clarified lysates (inputs) were analyzed by Western blotting with the indicated antibodies.

### The effect of BZLF1 and lytic infection on TRIM24/TRIM33/TRIM28 interactions

The effect of BZLF1 on TRIM protein interactions was assessed in two ways. AGS-BZLF1 cells were treated with 2 μg/ml doxycycline for 24 hours then lysed in RIPA buffer as above. 1.6 mg of clarified lysate was incubated at 4°C with mixing for 16 hours with 4 μg of anti-TRIM33 (PA5-82152; Invitrogen) or 4 μg negative control IgG (His antibody; sc-804, Santa Cruz) antibodies bound to protein A/G PLUS-Agarose beads (sc-2003, Santa Cruz). Agarose beads were then washed 4 times with 1 ml RIPA buffer and proteins were eluted in 2x SDS loading buffer. The eluates and 80 μg of clarified lysates (inputs) were analyzed by Western blotting with antibodies against TRIM33, TRIM24, TRIM28, BZLF1 and tubulin. In another approach, 293T cells were transfected with pPL17-BZLF1 or pCMV3FC empty vector and, 24 hours later, cells were lysed in 4 volumes of RIPA buffer. 1 mg of the clarified lysate was incubated at 4°C with mixing overnight with 4 μg of TRIM33 or negative control antibodies bound to protein A/G PLUS-Agarose beads. Agarose beads were then washed and eluted as above. The eluates and 80 μg of clarified lysates (inputs) were analyzed by Western blotting with the indicated antibodies.

To determine the TRIM24/TRIM33/TRIM28 interactions in lytic infection, NPC43-Z and Akata-Z cells were plated in ten 15cm dishes. Five dishes were treated with 2 μg/ml doxycycline for 24 hours, while five dishes were left untreated. Cells were then lysed in RIPA buffer as above and 4 mg of clarified lysate was incubated with 1 μg of anti-TRIM33 (Cell Signaling #90051) or 1 μg negative control IgG (Bethyl p120-101) antibodies at 4°C for 16 hours with mixing. The lysate/antibody mix was added to 50 μl of SureBeads Protein A Magnetic beads (BioRad #161–4013) pre-washed in RIPA buffer, and incubated 1 hour with mixing. Beads were then washed 4 times with 1 ml RIPA buffer and proteins were eluted in 2x SDS loading buffer. The eluates and 40 μg (1%) of clarified lysates (inputs) were analyzed by Western blotting with the indicated antibodies.

### Analysis of SUMOylation of the TRIM33 SUMO mutant

HEK293T cells were co-transfected with 0.5 μg of each pcDNA-6His-SUMO1 and pCMV3FC-TRIM33 expressing FLAG-tagged WT TRIM33 or TRIM SUMO mutant (SUMO^m^) using PolyJet (FroggaBio). 36 hours later, 10% of the cells were lysed in 2x SDS loading buffer to provide the input sample. 90% of cells were lysed in buffer G and 6His-tagged SUMO conjugates were purified as previously described [27]. Inputs and purified fractions were analyzed by Western blotting using anti-FLAG antibody.

### EBV genome quantification

AGS-EBV cells with TRIM33 KO, TRIM24 KO or control KO were harvested from 10-cm dishes and the total cellular and EBV DNA was extracted using the DNeasy Blood and Tissue Kit (Qiagen) following manufacturer’s protocol. 100 ng of DNA was used for genome amplification by polymerase chain reaction (PCR) using the Luna Universal qPCR Master Mix (New England BioLabs) with a reaction volume of 10 μl in a CFX384 real-time system (Bio-Rad). Primers used to quantify BZLF1 gene were described previously [93]. The relative EBV genome levels were obtained from 2−ΔΔCT using the comparative threshold cycle (C_T_) method and the amount of EBV genome copies in each sample were normalized to the amount of actin gene as control.

### BZLF1 transcripts levels upon TRIM loss

**A**GS-EBV cells with TRIM33 KO, TRIM24 KO or control KO were harvested from 10-cm dishes and total RNA was extracted using NucleoZOL (Macherey-Nagel) according to the manufacturer’s protocol. One microgram RNA was treated with 0.5 units of DNase I (New England BioLabs) for 15 minutes, followed by one-step real-time quantitative PCR (RT-qPCR) using the Luna Universal One-Step RT-qPCR kit (New England BioLabs) on 1/10^th^ of the DNase I-treated samples in a 10 μl reaction volume using the CFX384 real-time system (Bio-Rad). The primers used for BZLF1 transcript levels and the quantification analysis method was same as described above.

### Zp-luciferase reporter assays

AGS cells in 6-well dish were transfected with 1 μg of FLAG-TRIM33, FLAG-TRIM24 or pCMV3FC empty vector control along with 100 ng of pZp-luc reporter and 10 ng of pRL-promoterless (renilla control) plasmids using 9 μl PEI. Cells were harvested 24 hours post-transfection using passive lysis buffer (Promega) and assayed with the dual-luciferase reporter assay system (Promega). Luciferase readings were determined using the Lumat LB 9507 luminometer (Berthold Technologies). Firefly luciferase readings were normalized to renilla luciferase readings to compensate for transfection efficiencies.

### Effect of BZLF1 expression on TRIM protein levels

AGS-BZLF1 cells were treated with doxycycline for 24, 48 or 72 hours to induce BZLF1 expression or left untreated. Cells were lysed in 9M urea, sonicated and clarified by centrifugation. 60 μg of clarified lysate was analyzed by Western blotting with antibodies against TRIM33, TRIM24, TRIM28, BZLF1 and actin. Bands for TRIM33 and TRIM24 were quantified for three independent experiments using ImageJ, and average values and standard deviation was calculated.

### Effect of BZLF1 expression on TRIM33 SUMOylation

AGS-BZLF1 cells in 15 cm dishes were transfected with 12 μg of plasmid expressing 6His-SUMO2 [78] using PEI transfection reagent. 24 hours later, cells were treated with 2 μg/ml doxycycline (or left untreated) and harvested 24 hrs post-treatment. 10% of cells were lysed in 5x SDS loading buffer to provide the input sample. 90% of cells were lysed in buffer G (6 M guanidine hydrochloride, 10 mM Tris, 100 mM sodium phosphate, pH 8.0) and 6His-tagged SUMO conjugates were purified as previously described [27]. Inputs and purified fractions were analyzed by Western blotting with antibodies against TRIM33, BZLF1 and actin.

## Supporting information

S1 Table Mass Spectrometry data on SUMO-modified peptides. (XLSX)

S2 Table Positions of SUMO and phospho sites in SUMO-modified EBV Proteins. (DOCX)

S3 Table CRISPR guide oligonucleotides. (DOCX)

S1 Fig SUMO substrates with similar responses to EBV reactivation. A-H. Tabular summaries of the GG-K peptide and total protein data for a selection of SUMO substrates identified in the study as having multiple modification sites. Proteins are grouped broadly by similar responses to EBV reactivation. Only proteins with statistically significant changes are included. Cells are coloured by fold change with a thick border indicating statistical significance (p<0.05). Grey cells show proteins not detected in the whole cell extract samples.
(PDF)

S2 Fig Effect of EBV lytic infection on selected cellular protein levels. A. Western blots were performed on the cell lysates used for the SUMO proteomics experiments using the indicated antibodies. The levels of protein shown in are largely unaffected by EBV lytic infection. B. AGS cells containing the empty TRIPZ construct were treated with dox for 0, 24 or 48 hours, then whole cell lysates were analysed by Western blotting for TRIM24, TRIM33, SALL4 and actin (run on separate gels). The positions of SALL4A (*) and SALL4B (#) are marked. C. AGS-GFP cells were treated with dox and whole cell lysates analysed as in B. Western blots for GFP, TRIM24, TRIM33, SALL4 and actin are shown.
(TIF)

S3 Fig STRING interaction network for SUMO1 and SUMO2 substrates. A. Full interaction network generated by STRING (https://string-db.org/ - Stats; 717 nodes. 6269 edges. Average Node Degree 17.5. Expected edges 2371. PPI enrichment p-value<1r-16). Highlighted clusters were manually selected from a list of over 11500 enriched terms. B-C. Selected subnetworks. Data are averaged for SUMO1 and SUMO2 samples. The numbers of identified SUMO sites are indicated by node border thickness. Total GG-K peptide intensity per protein is indicated by node size (note log10 scale). Colour of nodes represent the rate of change in overall SUMOylation status (for all SUMO1 and SUMO2 sites in the protein) over the 24h period of EBV reactivation. Images were generated in Cytoscape.
(TIF)
