# Supplementary material for: Changes in SUMO-modified proteins in Epstein-Barr virus infection identifies reciprocal regulation of TRIM24/28/33 complexes and the lytic switch BZLF1
Source: PLoS Pathog. 2023 Jul 6;19(7):e1011477. doi: 10.1371/journal.ppat.1011477 (PMC10353822; doi:10.1371/journal.ppat.1011477)
Supplement: S2 Table — (DOCX) [file ppat.1011477.s002.docx]

**S2 Table. Positions of SUMO and Phospho sites in SUMO-modified EBV Proteins**

| **Protein name** | **Gene** | **Function** | **SUMO Site** | **Phospho site** |
| --- | --- | --- | --- | --- |
| EB1, Zebra, BZLF1 | BZLF1 | Zta transcriptional activator | K12*  K161* | T14* |
| Rta, BRLF1 | BRLF1 | Rta transcriptional activator. Downregulates cellular SUMOylation | K530* |  |
| EB2, SM, Mta | BSLF2/BMLF1 | mRNA binding and export. SUMO E3 ligase | K92* |  |
| EA-D, BMRF1 | BMRF1 | DNA polymerase processivity factor. Transcriptional activator. Inhibits DNA damage response. Promotes cellular SUMOylation | K212*  K228*  K380* | S314  S333  S335  S337  S349  S357  S384* |
| R1, BORF2 | BORF2 | Ribonucleotide-reductase, large subunit. Inhibits APOBEC3B antiviral activity | K741* |  |
| BALF2, SSDBP | BALF2 | ssDNA binding protein | K173* |  |
| LF2 | LF2 | Immune evasion | K250* |  |

*Identified in GlyGly-K IP samples. Remainder detected in crude cell lysates. All included phospho sites identified from peptides co-modified by SUMO (GG-K) had andromeda score >60 and mass error <1ppm. Further details of these sites, those EBV phospho sites not meeting these score and mass error cut-off values and cellular protein phosphorylation sites, can be found in S1 Table.
