# Supplementary material for: Changes in SUMO-modified proteins in Epstein-Barr virus infection identifies reciprocal regulation of TRIM24/28/33 complexes and the lytic switch BZLF1
Source: PLoS Pathog. 2023 Jul 6;19(7):e1011477. doi: 10.1371/journal.ppat.1011477 (PMC10353822; doi:10.1371/journal.ppat.1011477)
Supplement: S3 Table — (DOCX) [file ppat.1011477.s003.docx]

**S3 Table. CRISPR guide oligonucleotides**

| Target gene | Guide* | Forward sequence (5’ – 3’) | Reverse sequence (5’ – 3’) |
| --- | --- | --- | --- |
| Adeno-Associated Virus Integration Site 1 (AAVS1) | CG | cac cgG TCC CCT CCA CCC CAC AGT G | gaa cCA CTG TGG GGT GGA GGG GAC c |
| TRIM33 | E | cac cgG AAG ACA ATG CAA GTG CAG T | gaa cAC TGC ACT TGC ATT GTC TTC c |
|  | F | cac cgA TGT AGA GAC TGT CAG CTA T | gaa cAT AGC TGA CAG TCT CTA CAT c |
| TRIM24 | B | cac cgC GTG TAT CAG AGC TCA TCA G | gaa cCT GAT GAG CTC TGA TAC ACG c |
|  | C | cac cgT GTT AGA ACA TAA AGA GCA T | gaa cAT GCT CTT TAT GTT CTA ACA c |
|  | D | cac cgT CAG GTA TGT ACA AGC TGT G | gaa cCA CAG CTT GTA CAT ACC TGA c |

*Control guide (CG) targeting AAVS1 was reported in Mali et al. [1]; Guides E and F targeting TRIM33 and guides B, C and D targeting TRIM24 were reported in Sanjana et al.[2].

**References**

1. Mali P, Yang L, Esvelt KM, Aach J, Guell M, DiCarlo JE, et al. RNA-guided human genome engineering via Cas9. Science. 2013;339: 823-826.
2. Sanjana NE, Shalem O, Zhang F. Improved vectors and genome-wide libraries for CRISPR screening. Nat Methods. 2014;11: 783-784.
