# Supplementary material for: Changes in SUMO-modified proteins in Epstein-Barr virus infection identifies reciprocal regulation of TRIM24/28/33 complexes and the lytic switch BZLF1
Source: PLoS Pathog. 2023 Jul 6;19(7):e1011477. doi: 10.1371/journal.ppat.1011477 (PMC10353822; doi:10.1371/journal.ppat.1011477)
Supplement: S1 Fig — A-H. Tabular summaries of the GG-K peptide and total protein data for a selection of SUMO substrates identified in the study as having multiple modification sites. Proteins are grouped broadly by similar responses to EBV reactivation. Only proteins with statistically significant changes are included. Cells are coloured by fold change with a thick border indicating statistical significance (p<0.05). Grey cells show proteins not detected in the whole cell extract samples. (PDF) [file ppat.1011477.s004.pdf]

A

Cell fraction	GG-K Peptides (log$_2$ ratios v 0 hours)				Protein level (log$_2$ ratios v 0 hours)			
EBV reactivation time	12 hours		24 hours		12 hours		24 hours	
Cell type	SUMO1	SUMO2	SUMO1	SUMO2	SUMO1	SUMO2	SUMO1	SUMO2
SALL4-K156	2.22	0.90	2.73	0.97	0.31	1.43	2.62	3.22
SALL4-K316	5.21	-0.75	3.69	-0.53	0.31	1.43	2.62	3.22
SALL4-K372	3.46	-0.15	4.20	0.07	0.31	1.43	2.62	3.22
SALL4-K374	3.20	0.09	4.08	3.03	0.31	1.43	2.62	3.22
SALL4-K475	2.30	1.59	2.34	2.30	0.31	1.43	2.62	3.22
SALL4-K838	2.32	2.68	2.92	3.62	0.31	1.43	2.62	3.22
HHV4_BMRF1-K212	-0.40	-0.37	3.30	3.02	2.57	1.81	7.57	6.28
HHV4_BMRF1-K228	-0.55	0.05	2.38	2.35	2.57	1.81	7.57	6.28
HHV4_BMRF1-K380	-0.35	0.01	5.08	2.35	2.57	1.81	7.57	6.28
HHV4_BORF2-K741	-0.87	-0.06	1.76	3.26	0.87	2.82	10.05	8.93
HHV4_BRLF1-K530	-0.43	0.00	2.59	1.75	0.55	-0.61	4.88	3.87
HHV4_BSLF2/BMLF1-K92	0.12	0.15	2.86	2.64	4.98	3.95	8.30	6.91
HHV4_BZLF1-K12	6.01	3.77	4.64	7.77	8.58	8.27	8.73	8.59
HHV4_BZLF1-K161	1.79	2.21	4.47	5.13	8.58	8.27	8.73	8.59
HHV4_hyp.prot.-K250	0.38	1.32	0.50	3.38	1.24	2.32	7.26	7.41
HHV4_SSDBP-K173	-1.14	-0.66	2.61	3.71	3.16	2.86	9.28	7.54

SUMO and Protein level increase

B

Cell fraction	GG-K Peptides (log$_2$ ratios v 0 hours)				Protein level (log$_2$ ratios v 0 hours)			
EBV reactivation time	12 hours		24 hours		12 hours		24 hours	
Cell type	SUMO1	SUMO2	SUMO1	SUMO2	SUMO1	SUMO2	SUMO1	SUMO2
BRIX1-K31	-0.63	0.35	-1.25	0.20	0.51	-0.77	-1.01	-0.86
BRIX1-K314	-0.21	-0.33	-1.55	-1.47	0.51	-0.77	-1.01	-0.86
BRIX1-K322	-2.56	-0.08	-0.64	-2.01	0.51	-0.77	-1.01	-0.86
KLF5-K162	-0.78	-0.75	-2.97	-0.80	-0.10	-0.41	-1.80	-1.23
KLF5-K209	0.38	-1.08	-0.86	-4.21	-0.10	-0.41	-1.80	-1.23
TRIM24-K723	-0.67	-0.27	-0.93	-0.85	-0.54	-0.33	-1.15	-1.11
TRIM24-K741	-0.11	1.56	-1.08	0.47	-0.54	-0.33	-1.15	-1.11
TRIM24-K801	-1.48	-0.49	-2.23	-0.61	-0.54	-0.33	-1.15	-1.11
TRIM24-K992	-0.85	1.22	-1.32	0.26	-0.54	-0.33	-1.15	-1.11

SUMO and Protein level decrease

C

Cell fraction	GG-K Peptides (log$_2$ ratios v 0 hours)				Protein level (log$_2$ ratios v 0 hours)			
EBV reactivation time	12 hours		24 hours		12 hours		24 hours	
Cell type	SUMO1	SUMO2	SUMO1	SUMO2	SUMO1	SUMO2	SUMO1	SUMO2
BCLAF1-K437	0.79	-2.00	1.54	-2.70	0.24	-0.11	0.53	0.12
BCLAF1-K457	1.03	-0.66	0.00	-1.15	0.24	-0.11	0.53	0.12
BCLAF1-K491	0.18	1.00	0.03	1.00	0.24	-0.11	0.53	0.12
BCLAF1-K501	-1.33	-0.51	-1.39	-0.33	0.24	-0.11	0.53	0.12
BCLAF1-K548	0.04	-0.52	-0.07	-0.31	0.24	-0.11	0.53	0.12
BCLAF1-K580	-0.41	-0.11	-0.12	0.06	0.24	-0.11	0.53	0.12
BCLAF1-K676	0.18	0.51	0.93	0.16	0.24	-0.11	0.53	0.12
BCLAF1-K831	1.68	-0.07	0.12	-2.39	0.24	-0.11	0.53	0.12
TCOF1-K470	-0.52	-0.19	0.04	0.09	0.32	0.18	0.43	0.53
TCOF1-K600	-0.23	-0.26	0.48	0.28	0.32	0.18	0.43	0.53
TCOF1-K705	-0.70	-1.47	-1.19	-0.72	0.32	0.18	0.43	0.53
TCOF1-K732	-0.42	0.10	0.05	0.23	0.32	0.18	0.43	0.53
TCOF1-K755	-0.34	0.70	-0.06	0.60	0.32	0.18	0.43	0.53
TCOF1-K1238	0.95	-0.26	0.88	-0.78	0.32	0.18	0.43	0.53
TCOF1-K1414	-0.67	-0.53	-0.64	-0.13	0.32	0.18	0.43	0.53
SAFB2-K163	1.04	0.71	-0.62	-1.04	0.16	-0.76	0.30	-0.33
SAFB2-K199	1.17	-0.60	-0.99	-1.73	0.16	-0.76	0.30	-0.33
SAFB2-K225	0.20	0.10	-0.19	-0.43	0.16	-0.76	0.30	-0.33
SAFB2-K230	1.30	-0.14	1.21	-0.62	0.16	-0.76	0.30	-0.33
SAFB2-K252	-0.69	-0.03	-0.24	-0.48	0.16	-0.76	0.30	-0.33
SAFB2-K293	-1.59	-0.70	-2.12	0.75	0.16	-0.76	0.30	-0.33
SAFB2-K380	-0.48	-0.76	-0.70	-0.68	0.16	-0.76	0.30	-0.33
SAFB2-K385	-0.32	-0.30	-0.15	-0.96	0.16	-0.76	0.30	-0.33
SAFB2-K395	-0.10	-0.54	-0.24	-0.58	0.16	-0.76	0.30	-0.33
SAFB2-K517	1.54	-0.71	1.11	-0.89	0.16	-0.76	0.30	-0.33
SAFB2-K551	0.01	-0.20	0.32	-0.54	0.16	-0.76	0.30	-0.33
SAFB2-K586	-0.05	-0.01	-0.91	-0.15	0.16	-0.76	0.30	-0.33
NOLC1-K66	-0.85	0.15	-0.22	0.60	-0.18	-0.12	0.18	0.06
NOLC1-K75	-0.47	-0.27	-0.37	1.59	-0.18	-0.12	0.18	0.06
NOLC1-K185	-0.59	0.31	-0.39	0.86	-0.18	-0.12	0.18	0.06
NOLC1-K192	-0.47	-0.02	-0.24	-0.16	-0.18	-0.12	0.18	0.06
NOLC1-K405	-1.20	-0.29	-0.45	-0.37	-0.18	-0.12	0.18	0.06
NOLC1-K410	0.14	-0.37	-1.47	-0.37	-0.18	-0.12	0.18	0.06
NOLC1-K416	0.31	0.19	-1.29	0.89	-0.18	-0.12	0.18	0.06
NOLC1-K461	0.06	0.08	0.01	0.26	-0.18	-0.12	0.18	0.06
NOLC1-K588	-0.37	0.37	-0.19	0.83	-0.18	-0.12	0.18	0.06
NOLC1-K613	-0.10	-0.13	-0.04	0.15	-0.18	-0.12	0.18	0.06
NOLC1-K672	-1.78	-0.36	-1.07	0.43	-0.18	-0.12	0.18	0.06

Multi-Site Few significant changes

D

Cell fraction	GG-K Peptides (log$_2$ ratios v 0 hours)				Protein level (log$_2$ ratios v 0 hours)			
EBV reactivation time	12 hours		24 hours		12 hours		24 hours	
Cell type	SUMO1	SUMO2	SUMO1	SUMO2	SUMO1	SUMO2	SUMO1	SUMO2
RSL1D1-K29	-0.38	-0.39	-1.61	-1.23	0.20	-0.01	0.16	-0.05
RSL1D1-K120	0.00	-0.20	-2.37	-0.75	0.20	-0.01	0.16	-0.05
RSL1D1-K188	-0.25	-0.92	-0.50	-0.75	0.20	-0.01	0.16	-0.05
RSL1D1-K318	-0.39	-0.87	-1.08	-1.06	0.20	-0.01	0.16	-0.05
RSL1D1-K331	-0.62	-0.77	-0.24	-0.74	0.20	-0.01	0.16	-0.05
RSL1D1-K356	0.47	0.05	-0.43	-1.67	0.20	-0.01	0.16	-0.05
RSL1D1-K373	-0.05	-0.20	-0.50	-0.43	0.20	-0.01	0.16	-0.05
RSL1D1-K374	-1.85	-0.16	-1.90	0.18	0.20	-0.01	0.16	-0.05
RSL1D1-K380	0.42	-0.89	-0.10	-1.21	0.20	-0.01	0.16	-0.05
RSL1D1-K408	0.11	0.95	-1.28	0.71	0.20	-0.01	0.16	-0.05
RSL1D1-K417	0.24	0.52	-0.49	1.07	0.20	-0.01	0.16	-0.05
RSL1D1-K461	-0.09	-1.47	-0.85	-1.75	0.20	-0.01	0.16	-0.05
BEND3-K20	-0.86	-0.65	-0.81	-2.19				
BEND3-K73	-0.84	-0.55	0.04	-0.69				
BEND3-K129	0.23	0.01	-1.06	0.41				
BEND3-K142	-1.24	-1.69	-1.93	-0.79				
BEND3-K158	-1.20	-0.76	-1.40	-0.84				
BEND3-K512	-0.05	-0.67	-1.87	-2.97				
ZBTB25-K142	-1.22	-0.16	-1.59	-0.84				
ZBTB25-K198	-1.29	-0.93	-2.16	-0.72				
ZBTB25-K204	-1.55	0.34	-1.83	-1.13				
ZBTB2-K147	-2.61	-0.76	-2.35	-1.84				
ZBTB2-K154	-0.91	-0.61	-1.68	-1.19				
ZBTB2-K240	-0.11	-0.49	-0.48	-2.31				
ZBTB2-K304	-1.12	-0.58	-2.50	-1.24				
ZBTB2-K505	-0.01	-0.18	-0.27	-1.12				
RBM25-K578	-0.70	0.06	-0.97	0.49	0.15	-0.21	0.41	0.26
RBM25-K685	-0.99	1.13	-1.42	1.24	0.15	-0.21	0.41	0.26
RBM25-K697	1.27	-0.33	-1.71	0.05	0.15	-0.21	0.41	0.26
RBM25-K709	-1.89	-0.44	-1.37	-1.00	0.15	-0.21	0.41	0.26
RBM25-K722	-0.79	-1.73	-0.76	-1.39	0.15	-0.21	0.41	0.26
PML-K160	-2.79	0.28	-0.04	-2.08	-0.62	0.46	-0.46	0.63
PML-K426	0.11	-0.51	-0.45	-1.07	-0.62	0.46	-0.46	0.63
PML-K490	0.25	0.18	-0.64	-0.47	-0.62	0.46	-0.46	0.63
NOP58-K221	-0.80	-0.96	-1.23	-1.57	0.00	-0.38	0.29	-0.18
NOP58-K405	-0.25	-3.73	-2.78	-3.48	0.00	-0.38	0.29	-0.18
NOP58-K411	-0.12	-1.04	-0.88	-1.70	0.00	-0.38	0.29	-0.18
NOP58-K426	-0.78	-0.79	-1.45	-1.30	0.00	-0.38	0.29	-0.18
NOP58-K441	-0.40	-0.17	-1.59	-1.24	0.00	-0.38	0.29	-0.18
NOP58-K444	-0.29	-0.61	-0.30	-1.30	0.00	-0.38	0.29	-0.18
NOP58-K457	-1.18	0.33	-1.01	-0.23	0.00	-0.38	0.29	-0.18
NOP58-K465	0.02	-0.24	-1.06	-0.72	0.00	-0.38	0.29	-0.18
NOP58-K467	-0.08	-0.67	-1.81	-1.61	0.00	-0.38	0.29	-0.18
NOP58-K485	-0.44	0.85	-0.93	-2.35	0.00	-0.38	0.29	-0.18
NOP58-K497	-0.03	-0.37	-1.32	-0.53	0.00	-0.38	0.29	-0.18
BRD7-K21	-0.05	-0.55	-1.01	-1.09	-0.06	0.30	-0.04	0.88
BRD7-K28	0.13	-1.31	-1.39	-0.53	-0.06	0.30	-0.04	0.88
BRD7-K119	-0.85	0.37	-2.11	-1.06	-0.06	0.30	-0.04	0.88
BRD7-K127	-0.22	0.06	-1.13	-1.00	-0.06	0.30	-0.04	0.88
BRD7-K186	0.32	-1.10	-0.26	-0.68	-0.06	0.30	-0.04	0.88
BRD7-K197	0.28	-0.83	-0.46	0.99	-0.06	0.30	-0.04	0.88
BRD7-K212	0.28	-0.96	-0.81	-1.51	-0.06	0.30	-0.04	0.88
BRD7-K305	-0.06	0.01	-1.19	-0.58	-0.06	0.30	-0.04	0.88
BRD7-K307	-0.17	-0.32	-0.12	-0.85	-0.06	0.30	-0.04	0.88
BRD8-K362	-0.60	-0.79	-0.30	-1.19	-0.42	1.02	0.01	-0.41
BRD8-K503	-0.11	-0.52	-1.02	-0.92	-0.42	1.02	0.01	-0.41
BRD8-K569	-0.13	-0.41	-1.08	-1.16	-0.42	1.02	0.01	-0.41
BRD8-K606	0.04	-0.29	-0.84	-1.07	-0.42	1.02	0.01	-0.41
BRD8-K618	-0.98	-0.92	-1.84	-2.18	-0.42	1.02	0.01	-0.41
TOPORS-K73	-0.06	0.03	-0.78	-0.87				
TOPORS-K76	-0.25	-0.46	-1.42	0.02				
TOPORS-K83	-0.26	-0.63	-1.28	-0.87				
TOPORS-K88	-0.30	-0.26	-0.84	-0.28				
TOPORS-K819	0.65	-0.15	0.93	0.65				
TOPORS-K837	-0.55	0.04	-0.41	-0.90				
TOPORS-K950	0.70	0.91	-1.03	0.62				

Losing SUMO

E

Cell fraction	GG-K Peptides (log$_2$ ratios v 0 hours)				Protein level (log$_2$ ratios v 0 hours)			
EBV reactivation time	12 hours		24 hours		12 hours		24 hours	
Cell type	SUMO1	SUMO2	SUMO1	SUMO2	SUMO1	SUMO2	SUMO1	SUMO2
RBBP4-K160	0.21	0.20	0.07	1.02	0.01	0.21	0.40	0.39
RBBP5-K397	0.51	0.16	2.86	-0.21	-0.16	-0.23	-0.18	0.06
RBBP7-K25	2.19	0.93	3.77	2.42	1.00	0.50	0.82	0.68
RBBP7-K150	1.47	0.72	2.07	2.72	1.00	0.50	0.82	0.68
RBBP7-K297	0.66	0.81	1.06	1.60	1.00	0.50	0.82	0.68
SUMO1-K7	0.51	0.22	1.50	1.39	0.01	-0.08	0.17	0.05
SUMO1-K16	1.53	0.26	1.59	0.86	0.01	-0.08	0.17	0.05
SUMO1-K17	0.55	2.00	2.24	2.82	0.01	-0.08	0.17	0.05
SUMO1-K23	0.70	0.85	1.60	2.92	0.01	-0.08	0.17	0.05
SUMO1-K25	0.49	0.28	1.21	0.81	0.01	-0.08	0.17	0.05
SUMO1-K37	1.30	0.46	2.95	2.36	0.01	-0.08	0.17	0.05
SUMO1-K39	1.22	0.16	1.97	0.39	0.01	-0.08	0.17	0.05
SUMO1-K78	1.08	1.16	0.71	2.72	0.01	-0.08	0.17	0.05
SUMO2-K5	0.05	0.85	0.92	3.07	-0.57	0.90	0.44	0.21
SUMO2-K7	0.60	2.59	1.29	3.81	-0.57	0.90	0.44	0.21
SUMO2-K11	1.00	0.98	2.07	1.85	-0.57	0.90	0.44	0.21
SUMO2-K21	1.58	0.04	2.28	1.20	-0.57	0.90	0.44	0.21
SUMO2-K33	1.17	0.05	1.88	0.92	-0.57	0.90	0.44	0.21
SUMO3-K7	0.27	0.13	0.15	1.97	-0.20	0.04	0.72	-0.01
SUMO3-K11	0.40	0.90	1.60	1.99	-0.20	0.04	0.72	-0.01
SUMO3-K20	-0.12	-0.26	-0.27	0.37	-0.20	0.04	0.72	-0.01
SUMO3-K32	1.18	0.05	1.87	0.93	-0.20	0.04	0.72	-0.01
SUMO3-K42	0.23	0.66	1.80	1.63	-0.20	0.04	0.72	-0.01
NSMCE2-K30	0.99	0.84	2.15	2.42				
NSMCE2-K65	2.03	1.51	2.21	1.66				
NSMCE2-K113	1.67	0.73	0.87	0.78				
NSMCE4A-K382	1.73	1.37	2.39	1.56				
RBM14-K126	0.81	0.83	1.68	3.03	0.32	0.28	0.70	0.32
RBM14-K135	0.75	1.97	0.86	1.72	0.32	0.28	0.70	0.32
RBM14-K140	1.33	0.53	1.28	1.13	0.32	0.28	0.70	0.32
RBM14-K149	1.90	0.55	3.05	3.92	0.32	0.28	0.70	0.32
NFATC2-K682	2.01	-0.37	4.30	6.42	-0.23	0.28	0.23	0.18
NFATC2-K895	2.40	1.32	4.45	3.50	-0.23	0.28	0.23	0.18
CHD4-K45	-0.10	0.44	1.23	0.52	-0.03	-0.03	0.09	0.03
CHD4-K922	0.33	0.94	1.52	1.50	-0.03	-0.03	0.09	0.03
CHD4-K956	0.47	0.03	2.05	1.31	-0.03	-0.03	0.09	0.03
CHD4-K974	1.17	1.61	2.04	0.57	-0.03	-0.03	0.09	0.03
CHD4-K997	0.84	2.44	2.02	3.43	-0.03	-0.03	0.09	0.03
CHD4-K1020	0.49	1.13	0.76	1.32	-0.03	-0.03	0.09	0.03
GATAD2A-K27	0.84	2.12	1.66	3.08	-0.18	-0.20	0.21	0.12
GATAD2A-K93	-0.21	1.42	3.81	0.91	-0.18	-0.20	0.21	0.12
GATAD2A-K487	0.73	0.52	2.02	3.62	-0.18	-0.20	0.21	0.12
GATAD2A-K499	1.02	-0.26	1.88	-0.50	-0.18	-0.20	0.21	0.12
GATAD2B-K33	0.11	-0.17	2.21	3.89	0.11	0.07	0.32	0.38
GATAD2B-K337	1.29	1.74	2.70	2.33	0.11	0.07	0.32	0.38
GATAD2B-K438	0.46	-0.75	2.64	1.87	0.11	0.07	0.32	0.38
GATAD2B-K482	1.57	0.62	1.90	1.17	0.11	0.07	0.32	0.38
SP100-K241	-0.52	0.60	1.63	2.15				
SP100-K297	0.97	1.22	1.06	2.13				
SP100-K306	0.20	0.28	1.81	1.85				
SERBP1-K91	0.07	-0.15	2.22	1.61	-0.45	-0.56	-1.07	-0.20
SERBP1-K102	-0.63	-0.12	1.71	2.37	-0.45	-0.56	-1.07	-0.20
SERBP1-K122	-0.75	0.71	1.54	3.04	-0.45	-0.56	-1.07	-0.20
SERBP1-K228	-1.94	0.65	-0.06	0.79	-0.45	-0.56	-1.07	-0.20
SERBP1-K281	0.00	0.48	1.56	4.00	-0.45	-0.56	-1.07	-0.20
PARP1-K203	0.72	0.51	2.38	3.51	0.08	-0.11	0.14	0.22
PARP1-K249	2.76	-1.55	3.01	-0.57	0.08	-0.11	0.14	0.22
PARP1-K467	-0.16	0.09	2.65	1.56	0.08	-0.11	0.14	0.22
PARP1-K486	0.33	0.26	2.69	1.81	0.08	-0.11	0.14	0.22
PARP1-K512	0.46	-1.31	2.20	4.44	0.08	-0.11	0.14	0.22
SAMHD1-K469	0.47	0.64	2.39	2.83	0.15	-0.03	-0.31	-0.30
SAMHD1-K622	0.30	0.86	2.19	2.78	0.15	-0.03	-0.31	-0.30

Gaining SUMO

F

Cell fraction / EBV reactivation time / Cell type	GG-K Peptides (log_2 ratios v 0 hours) 12 hours SUMO1	12 hours SUMO2	24 hours SUMO1	24 hours SUMO2	Protein level (log_2 ratios v 0 hours) 12 hours SUMO1	12 hours SUMO2	24 hours SUMO1	24 hours SUMO2
RGPD1-K1621	0.05	0.42	2.14	0.19				
RGPD1-K2181	0.10	0.17	2.32	0.15				
RGPD1-K2197	0.07	-0.31	3.23	0.29				
RGPD1-K2212	-0.88	0.18	2.78	0.10				
RGPD1-K2581	-0.73	0.19	2.52	-0.01				
RGPD1-K2592	0.85	0.58	1.74	3.42				
RGPD4-K1596	-0.08	0.74	2.46	3.36				
AHNAK-K530	0.19	-0.03	1.52	0.16	0.17	0.06	0.01	0.03
AHNAK-K712	0.49	-0.39	2.00	0.25	0.17	0.06	0.01	0.03
AHNAK-K833	-0.61	0.13	1.24	1.87	0.17	0.06	0.01	0.03
AHNAK-K891	0.68	-0.62	4.85	-0.71	0.17	0.06	0.01	0.03
AHNAK-K961	-0.19	1.24	0.58	2.68	0.17	0.06	0.01	0.03
AHNAK-K1488	-0.57	0.01	0.08	-0.08	0.17	0.06	0.01	0.03
AHNAK-K2132	0.11	0.15	1.56	1.53	0.17	0.06	0.01	0.03
AHNAK-K3164	-0.62	0.33	1.98	0.77	0.17	0.06	0.01	0.03
AHNAK-K3201	0.91	0.11	3.99	0.33	0.17	0.06	0.01	0.03
RANBP2-K1350	1.06	0.37	2.95	1.79	0.10	0.06	0.16	0.17
RANBP2-K1414	0.95	1.01	2.89	2.44	0.10	0.06	0.16	0.17
RANBP2-K1522	0.46	-0.04	3.33	-0.18	0.10	0.06	0.16	0.17
RANBP2-K1542	0.01	0.42	3.25	1.29	0.10	0.06	0.16	0.17
RANBP2-K1593	0.25	0.32	1.47	0.84	0.10	0.06	0.16	0.17
RANBP2-K1605	0.04	0.23	2.32	2.35	0.10	0.06	0.16	0.17
RANBP2-K1851	-0.53	0.05	2.26	-0.01	0.10	0.06	0.16	0.17
RANBP2-K2529	-0.39	0.03	2.54	-0.38	0.10	0.06	0.16	0.17
RANBP2-K2571	-0.08	0.45	2.46	2.75	0.10	0.06	0.16	0.17
RANBP2-K2612	0.07	-0.45	2.15	-0.12	0.10	0.06	0.16	0.17
RANBP2-K2792	0.07	0.03	2.00	2.63	0.10	0.06	0.16	0.17
RANBP2-K2801	-0.47	-0.11	2.86	0.17	0.10	0.06	0.16	0.17
RANBP2-K2815	0.12	-0.50	2.95	0.06	0.10	0.06	0.16	0.17
RANBP2-K2823	-0.32	-0.09	3.09	0.22	0.10	0.06	0.16	0.17
RANBP2-K2866	1.78	0.73	4.57	0.54	0.10	0.06	0.16	0.17
RANBP2-K2924	-0.35	-0.42	2.93	0.35	0.10	0.06	0.16	0.17
RANBP2-K3025	0.30	-0.50	2.47	-0.35	0.10	0.06	0.16	0.17
RANGAP1-K8	3.32	0.13	5.87	0.37	-0.06	0.21	-0.57	-0.98
RANGAP1-K15	-0.10	0.28	2.85	0.57	-0.06	0.21	-0.57	-0.98
RANGAP1-K26	0.12	-0.73	4.03	-0.23	-0.06	0.21	-0.57	-0.98
RANGAP1-K39	0.11	0.20	2.02	-0.18	-0.06	0.21	-0.57	-0.98
RANGAP1-K445	-0.74	0.00	1.73	0.12	-0.06	0.21	-0.57	-0.98
RANGAP1-K452	1.49	0.43	3.24	-0.25	-0.06	0.21	-0.57	-0.98
SP1-K9	-0.65	-1.19	2.56	-0.19	0.24	1.98	0.73	1.71
SP1-K16	-0.63	-1.35	2.54	-0.19	0.24	1.98	0.73	1.71

SUMO1 specific changes

G

Cell fraction / EBV reactivation time / Cell type	GG-K Peptides (log_2 ratios v 0 hours) 12 hours SUMO1	12 hours SUMO2	24 hours SUMO1	24 hours SUMO2	Protein level (log_2 ratios v 0 hours) 12 hours SUMO1	12 hours SUMO2	24 hours SUMO1	24 hours SUMO2
ZNF451-K102	-0.19	-0.95	-0.56	-1.08	0.91	0.64	-0.49	0.26
ZNF451-K106	-2.01	-0.14	-0.76	-1.51	0.91	0.64	-0.49	0.26
ZNF451-K288	-0.39	-0.18	0.52	1.60	0.91	0.64	-0.49	0.26
ZNF451-K706	1.69	-0.11	-0.30	-0.13	0.91	0.64	-0.49	0.26
ZNF451-K779	0.69	0.12	0.63	0.45	0.91	0.64	-0.49	0.26
ZNF451-K817	1.54	0.39	1.60	0.80	0.91	0.64	-0.49	0.26
ZNF451-K827	-1.13	0.10	1.03	3.52	0.91	0.64	-0.49	0.26
ZNF451-K832	-0.62	0.16	-0.98	0.92	0.91	0.64	-0.49	0.26
ZNF451-K843	0.19	0.23	0.79	1.35	0.91	0.64	-0.49	0.26
ZNF451-K845	0.20	0.61	0.10	2.27	0.91	0.64	-0.49	0.26
ZNF451-K993	-0.72	-1.06	0.39	0.84	0.91	0.64	-0.49	0.26
SMC5-K293	-0.49	3.12	0.23	3.96	-0.02	-0.02	0.05	-0.38
SMC5-K911	-0.73	2.72	-0.81	3.41	-0.02	-0.02	0.05	-0.38

SUMO2 specific changes

H

Cell fraction / EBV reactivation time / Cell type	GG-K Peptides (log_2 ratios v 0 hours) 12 hours SUMO1	12 hours SUMO2	24 hours SUMO1	24 hours SUMO2	Protein level (log_2 ratios v 0 hours) 12 hours SUMO1	12 hours SUMO2	24 hours SUMO1	24 hours SUMO2
TRIM28-K199	0.56	-0.46	-0.15	-0.02	-0.09	0.00	-0.15	-0.47
TRIM28-K289	-0.99	-0.11	-0.22	0.81	-0.09	0.00	-0.15	-0.47
TRIM28-K469	0.77	2.48	1.86	2.43	-0.09	0.00	-0.15	-0.47
TRIM28-K484	2.03	0.12	3.55	-0.68	-0.09	0.00	-0.15	-0.47
TRIM28-K507	-0.28	-0.45	0.10	-0.03	-0.09	0.00	-0.15	-0.47
TRIM28-K554	1.03	-0.47	-0.58	-0.48	-0.09	0.00	-0.15	-0.47
TRIM28-K575	-1.14	-0.36	-0.17	-0.96	-0.09	0.00	-0.15	-0.47
TRIM28-K676	2.92	-0.39	3.38	0.75	-0.09	0.00	-0.15	-0.47
TRIM28-K750	-0.28	-0.15	-0.36	-0.86	-0.09	0.00	-0.15	-0.47
TRIM28-K779	-1.88	-0.80	2.07	1.18	-0.09	0.00	-0.15	-0.47
TRIM28-K804	-1.68	1.55	-0.57	3.35	-0.09	0.00	-0.15	-0.47
MGA-K349	-0.30	0.28	-0.46	-0.26	0.01	-0.92	-0.54	-0.18
MGA-K403	-1.00	0.21	-0.75	1.32	0.01	-0.92	-0.54	-0.18
MGA-K570	0.72	0.67	0.31	-0.21	0.01	-0.92	-0.54	-0.18
MGA-K654	0.93	0.23	-0.28	-0.37	0.01	-0.92	-0.54	-0.18
MGA-K1946	2.28	0.82	1.81	0.87	0.01	-0.92	-0.54	-0.18
MGA-K2074	0.85	1.15	0.32	0.70	0.01	-0.92	-0.54	-0.18
MGA-K2096	0.98	-0.19	1.58	0.12	0.01	-0.92	-0.54	-0.18
MGA-K2590	1.56	0.56	1.07	-0.28	0.01	-0.92	-0.54	-0.18
TRIM33-K769	2.13	0.37	1.99	0.69	0.26	-0.18	0.00	0.07
TRIM33-K774	2.04	0.20	2.26	0.68	0.26	-0.18	0.00	0.07
TRIM33-K776	1.27	1.60	1.11	2.90	0.26	-0.18	0.00	0.07
TRIM33-K793	0.84	2.09	0.64	3.19	0.26	-0.18	0.00	0.07

Varied site-specific responses

Not detected

-8 -6 -4 -2 0 2 4 6 8

Log_2 Ratio

t-test p<0.05
