# Supplementary figures and images for: Changes in SUMO-modified proteins in Epstein-Barr virus infection identifies reciprocal regulation of TRIM24/28/33 complexes and the lytic switch BZLF1
Source: PLoS Pathog. 2023 Jul 6;19(7):e1011477. doi: 10.1371/journal.ppat.1011477 (PMC10353822; doi:10.1371/journal.ppat.1011477)

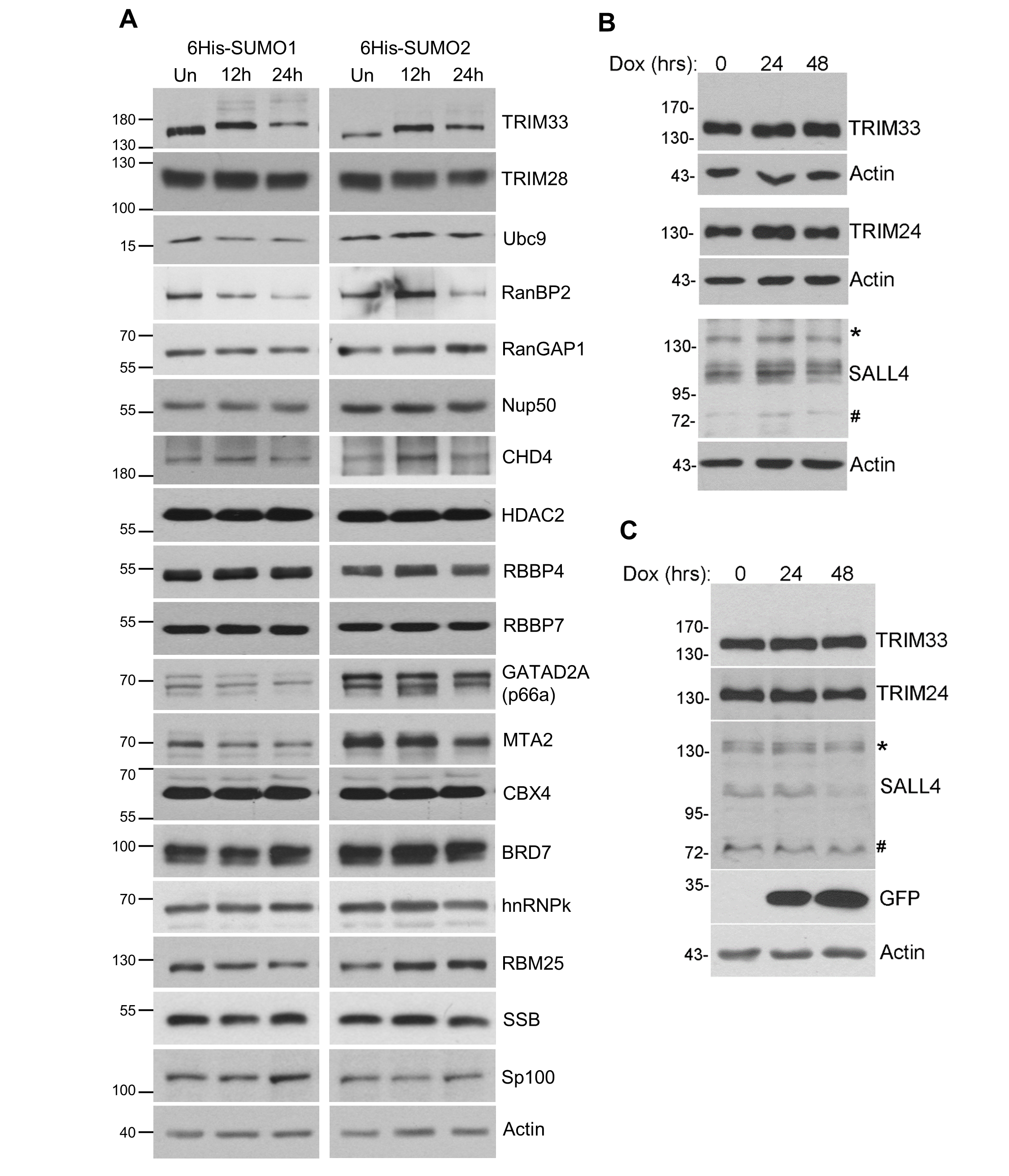

Supplement: S2 Fig — A. Western blots were performed on the cell lysates used for the SUMO proteomics experiments using the indicated antibodies. The levels of protein shown in are largely unaffected by EBV lytic infection. B. AGS cells containing the empty TRIPZ construct were treated with dox for 0, 24 or 48 hours, then whole cell lysates were analysed by Western blotting for TRIM24, TRIM33, SALL4 and actin (run on separate gels). The positions of SALL4A (*) and SALL4B (#) are marked. C. AGS-GFP cells were treated with dox and whole cell lysates analysed as in B. Western blots for GFP, TRIM24, TRIM33, SALL4 and actin are shown. (TIF) [file ppat.1011477.s005.tif]

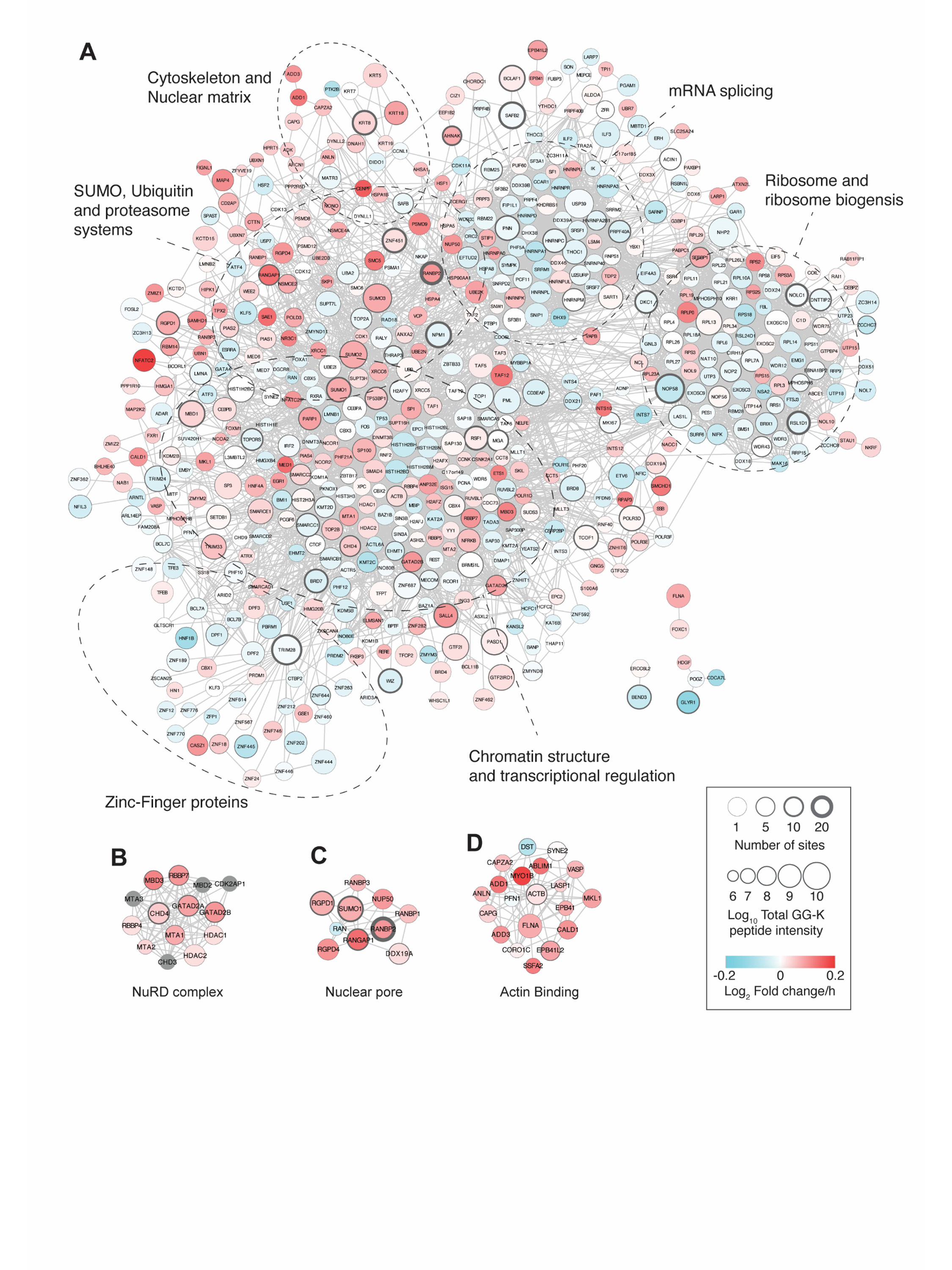

Supplement: S3 Fig — A. Full interaction network generated by STRING (https://string-db.org/ - Stats; 717 nodes. 6269 edges. Average Node Degree 17.5. Expected edges 2371. PPI enrichment p-value<1r-16). Highlighted clusters were manually selected from a list of over 11500 enriched terms. B-C. Selected subnetworks. Data are averaged for SUMO1 and SUMO2 samples. The numbers of identified SUMO sites are indicated by node border thickness. Total GG-K peptide intensity per protein is indicated by node size (note log10 scale). Colour of nodes represent the rate of change in overall SUMOylation status (for all SUMO1 and SUMO2 sites in the protein) over the 24h period of EBV reactivation. Images were generated in Cytoscape. (TIF) [file ppat.1011477.s006.TIF]
